# Predictive Tires for Electric and Autonomous Vehicles: A Comprehensive Review

**DOI:** 10.3390/s26206477

**Published:** 2026-10-13

**Authors:** Constantin-Cosmin Danci, Nicolae Burnete, Adrian Todoruț, Nicolae Cordoș, Irina Duma, István Barabás, Alexandru Terec

**Affiliations:** Department of Automotive Engineering and Transports, Technical University of Cluj-Napoca, 400641 Cluj-Napoca, Romania; cosmin.danci@auto.utcluj.ro (C.-C.D.); nicolae.burnete@auto.utcluj.ro (N.B.); adrian.todorut@auto.utcluj.ro (A.T.); nicolae.cordos@auto.utcluj.ro (N.C.); istvan.barabas@auto.utcluj.ro (I.B.); alexandru.terec@auto.utcluj.ro (A.T.)

**Keywords:** predictive tires, vehicle dynamics, electric vehicles, autonomous vehicles, smart sensors

## Abstract

The role of tires in vehicle dynamics has evolved from passive support elements to active sources of information. This paper provides a comprehensive synthesis of recent advances in wheel–road interaction, structural optimization, and intelligent sensing, toward the emerging concept of the predictive tire. Fundamental phenomena, including slip-induced energy losses, aquaplaning thresholds, and radial stiffness variation, are analyzed for their impact on safety, ride comfort, and energy efficiency. Structural developments, including multi-objective optimizations and advanced composite materials targeting rolling-resistance reduction and NVH (Noise, Vibration, Harshness) improvement, are important for electric-vehicle applications. At the same time, progress is being made in the integration of sensors, such as polyvinylidene fluoride piezoelectric films (PVDF), distributed strain sensors, embedded accelerometers, and hybrid tire pressure monitoring systems (h-TPMSs), along with advanced data-driven methods such as fuzzy logic, hybrid Kalman filters, and Transformer neural networks for estimating friction coefficients and contact forces. The integration of these directions supports the tire’s transition toward a predictive multifunctional platform supplying real-time variables for the anti-lock braking system (ABS), electronic stability control (ESC), torque vectoring, and advanced driver assistance systems (ADASs), reinforcing its role in the safe and intelligent mobility of electric and autonomous vehicles.

## 1. Introduction

The tire is the only point of contact between the vehicle and the road surface [1,2,3,4,5], and its characteristics directly determine the transmission of longitudinal, lateral, and vertical forces, influencing safety, stability, and energy efficiency [6,7,8,9]. In vehicle dynamics, tires play a dual role: one related to fundamental traction and braking performance, and another as a determining factor in vibro-acoustic comfort (Noise, Vibration, Harshness, NVH), durability, and extreme maneuvering [6,10,11].

For electric and autonomous vehicles, these roles become even more critical, as the vehicle’s mass exceeds that of conventional vehicles of the same class and the demands on range and reliability are higher [12,13,14,15,16]. As a result, the tire must simultaneously meet contradictory requirements, such as high grip and stability, but also low rolling resistance and real-time road-condition information [17].

Recent literature has addressed individual dimensions of intelligent tire research with increasing depth. In ref. [18], in-tire sensing systems and tire–road contact state-estimation methods for autonomous vehicle applications are surveyed, proposing design strategies for sensing systems adapted to the harsh operational conditions of tires. In ref. [19], tire–road friction coefficient-estimation methods are reviewed across three categories, off-board sensor-based, vehicle dynamics-based, and data-driven approaches, providing a comparative analysis of their respective strengths and limitations. In ref. [20], smart tires are examined as real-time friction monitoring tools from a road safety and infrastructure perspective, highlighting current challenges and future research prospects in the context of autonomous driving mobility patterns. In ref. [21], a comprehensive comparative review of tire-pressure monitoring methods developed over the past two decades for intelligent vehicles is provided, covering indirect TPMS approaches and their applicability under complex operating conditions. Paper [22] introduced a novel bench-to-road testing framework for intelligent tire development, explicitly identifying the absence of integrated validation protocols as a critical barrier to large-scale deployment. In ref. [23], a hierarchical friction coefficient estimator for four motorized wheel electric vehicles combining an Unscented Kalman Filter with a General Regression Neural Network is proposed, demonstrating the feasibility of sensor-free estimation under both low and high excitation conditions. A comparative overview of the thematic coverage of these existing reviews relative to the present work is provided in Table 1.

Despite these valuable contributions, a gap persists in the literature: to the best of our knowledge, none of the reviews discussed above simultaneously synthesizes wheel–road interaction phenomenology, structural and composite material optimization, distributed sensing architectures, and multimodal friction coefficient estimation within a unified predictive tire framework. In particular, the cross-domain interdependencies between these directions—wherein structural parameters constrain sensor integration, material properties influence estimation accuracy, and sensing outputs that determine the performance boundaries of control systems—appear not to have been jointly analyzed.

In this review, a tire system is classified as predictive only if it satisfies four minimum criteria: (i) distributed or embedded sensing capable of measuring at least one tire–road interaction variable at the tire–road interface; (ii) real-time estimation of a state or parameter not directly measurable by the sensor itself, such as the friction coefficient or vertical load; (iii) an anticipatory capability, whereby the estimation is used to infer an imminent condition, such as loss of adhesion or aquaplaning onset, rather than only reporting the current state; and (iv) a defined interface to a vehicle-level control system, such as the anti-lock braking system (ABS), electronic stability control (ESC), torque vectoring, or advanced driver-assistance systems (ADASs). This distinguishes the predictive tire from the broader categories of “smart tire” (criterion i only) and “sensor-equipped tire” (criteria i-ii, without iii-iv), which are used interchangeably in much of the surveyed literature.

This lack of integration limits the transferability of isolated advances to the practical design constraints of electric and autonomous vehicles, where all four domains must function as an integrated whole. Building on the operational definition above, the present work makes three concrete contributions that differentiate it from the closest existing reviews [18,19,20,21,22,23], summarized in Table 1. First, it provides a cross-domain synthesis explicitly linking tire–road interaction, structural optimization, intelligent sensing, and multimodal tire–road friction coefficient (TRFC) estimation, substantiated through three worked interdependency cases (radial force variation, aquaplaning, and low-adhesion TRFC estimation) presented in Section 6, rather than treating the four domains as independently reviewed topics. Second, it introduces the operational definition above as the basis for the comparative analysis in Table 1, which indicates that, among the six closest reviews, none jointly addresses cross-domain interdependencies. Third, it connects the resulting predictive-tire framework explicitly to vehicle-level outcomes, energy efficiency and range for electric vehicles, and perception redundancy for autonomous driving systems, discussed in Section 7, rather than reporting tire-level performance metrics in isolation.

### 1.1. The Importance of Tires for Road Safety and Performance

Excessive tire wear represents a significant risk to road safety by reducing traction and increasing braking distance, especially on wet surfaces [24,25,26]. According to crash data from the National Highway Traffic Safety Administration (NHTSA) [27], about 26% of tires with a tread depth of 1.6 mm or less were on vehicles that experienced tire problems in the pre-crash phase, versus 8% of tires with a tread depth of 2.4 ÷ 3.2 mm, which highlights the need for proactive wear monitoring. At the same time, the tire, as the only point of contact with the road, is crucial in managing critical phenomena such as skidding, aquaplaning, or sudden loss of traction [28,29,30].

The performance of a tire is determined by its composite structure and complex interaction with the road surface. The components of the tire (sidewall, tread, carcass) influence the dynamic response [31], which has led to the emergence of specialized sensor systems for tread-block force measurement [32], friction estimation [33] or contact patch-size measurement [34]. These measurements are not only useful for monitoring tire characteristics, but also for providing critical data needed for predictive control algorithms.

#### 1.1.1. Smart Tires and Active Control Systems

An emerging trend in recent years is the shift from passive tires to smart tires, capable of transmitting information about their own condition and the quality of the road surface. The integration of 3D Micro-Electro-Mechanical Systems (MEMSs) accelerometers, piezoelectric Polyvinylidene Fluoride (PVDF) sensors, strain sensors, or force-sensitive resistors allows real-time data to be obtained on vertical load, contact patch size, friction coefficient, or slip angle [35,36,37].

This capability is essential for the operation of the Anti-lock Braking System (ABS), Electronic Stability Control (ESC), traction control, or torque vectoring, which, in the absence of direct measurements, rely on indirect estimates with limited accuracy [38,39,40,41,42,43,44]. The concept of smart tire also extends the tire’s role to that of a redundant sensor for Advanced Driver Assistance Systems (ADASs) and autonomous vehicles (AVs), providing information to Light Detection and Ranging (LiDAR), radar, or camera systems.

#### 1.1.2. The Relevance of Using Predictive Tires for Electric and Autonomous Vehicles

Electric vehicles amplify the role of tires through three particular aspects: higher mass, instant torque, and increased range requirements [12,35,45]. Rolling resistance, which accounted for about 24 ÷ 37% of the energy consumption of the electric vehicle simulated in [16] at 80 km/h (between 0 and −30 °C), becomes a central target for optimization [16,35,41,46,47,48], with its standardized measurement governed by ISO 28580:2018 [49], which defines steady-state single-point testing conditions to ensure inter-laboratory comparability across tire-development programs.

At the same time, the optimized materials and designs used for electric-vehicle tires must reduce viscoelastic losses without compromising grip and durability [15,50]. Consequently, tire compound development for electric vehicles requires an engineered balance between the loss modulus at high temperatures, which governs rolling resistance, and the loss modulus at low temperatures, which determines cold grip—two properties that are inherently conflicting within conventional polymer networks [13].

Architectures with motors located in the wheel or Four-Wheel Independent Driving (4WID)/Four-Wheel Independent Steering (4WIS) types allow for high torque control [50,51,52,53,54,55,56,57], but this advantage depends on the accurate estimation of the TRFC and the forces transmitted to each wheel [43,58]. In the case of autonomous vehicles, information about road conditions and the friction coefficient becomes indispensable for planning safe trajectories, especially in rainy, snowy, or icy conditions [59,60,61,62].

#### 1.1.3. Tire–Road Friction Coefficient (TRFC) Used as a Critical Parameter

The TRFC defines the absolute limit of the longitudinal and lateral tangential forces that can be generated by the tire, and therefore the boundary of the vehicle’s dynamic stability [53,63]. Its accurate estimation is a fundamental condition for optimizing the performance of active systems such as ABS, ESC, advanced emergency braking (AEB), or torque vectoring. Recent literature shows that model-based approaches, such as Kalman observers, interacting multiple models (IMMs) or proportional multiple integration (PMI), and data-driven approaches (neural networks, including Transformer-based architectures, as well as image and vehicle data sensor fusion) are moving toward increasingly robust solutions [64,65,66,67]. Transformer models, based on self-attention mechanisms, enable the efficient processing of temporal sequences of vehicle data by capturing long-range dependencies, which improves the estimation of the tire–road friction coefficient under rapidly varying driving conditions. Conversely, the absence of this information forces the control to be set conservatively, with significant penalties on performance and efficiency [68].

### 1.2. Review Methodology

This review adopts the Preferred Reporting Items for Systematic Reviews and Meta-Analyses (PRISMA) 2020 reporting principles, adapted to a structured narrative review of heterogeneous engineering literature spanning materials characterization, sensor technology, and estimation algorithms. No protocol was registered in advance, and screening was carried out by a single reviewer rather than independently in duplicate. Formal risk-of-bias assessment tools, designed for the synthesis of clinical-trial evidence, were not applicable to this literature and were therefore not used. All other PRISMA 2020 reporting principles [69] were followed throughout, to keep the selection process transparent and reproducible.

Three databases were queried: ScienceDirect, Web of Science (Core Collection), and IEEE Xplore. The search window was restricted to publications between 2014 and 2026, with particular emphasis on work published from 2019 onwards. The literature search was carried out between July 2025 and April 2026. The record counts reported in this section and in Table A1 (Appendix A) correspond to the queries as executed during this period. Re-running the same queries at a later date will return higher counts, owing to continued publication activity in this rapidly developing field. The complete search strings, the filter applied, and the number of records returned by each database are reported in Table A1. The keyword groups covered the four thematic domains of this review: wheel–road interaction (“tire–road friction”, “wheel–road contact”, “aquaplaning”, “slip energy”), structural optimization (“tire structural optimization”, “radial stiffness”, “NVH tire”, “composite tire material”), intelligent sensing (“smart tire”, “intelligent tire”, “tire sensor”, “PVDF tire”, “FBG tire”, “TPMS”), and friction estimation (“tire–road friction coefficient estimation”, “TRFC”, “Kalman filter tire”, “neural-network friction”). For IEEE Xplore, the results were restricted to journal articles and early-access articles, conference proceedings being excluded at this stage in order to keep the corpus consistent with the peer-review level of the other two databases.

Studies were included if they addressed at least one of the four thematic domains in the context of road vehicles; reported quantitative experimental or simulation results, or a validated methodological framework; and were published in English in a peer-reviewed venue. Studies were excluded if they addressed exclusively non-automotive tire applications, such as aircraft or bicycle tires; dealt with tire manufacturing or chemical synthesis without mechanical characterization; provided insufficient methodological detail for meaningful cross-study comparison; or had been superseded by a more complete journal version of the same work.

**Figure 1:**
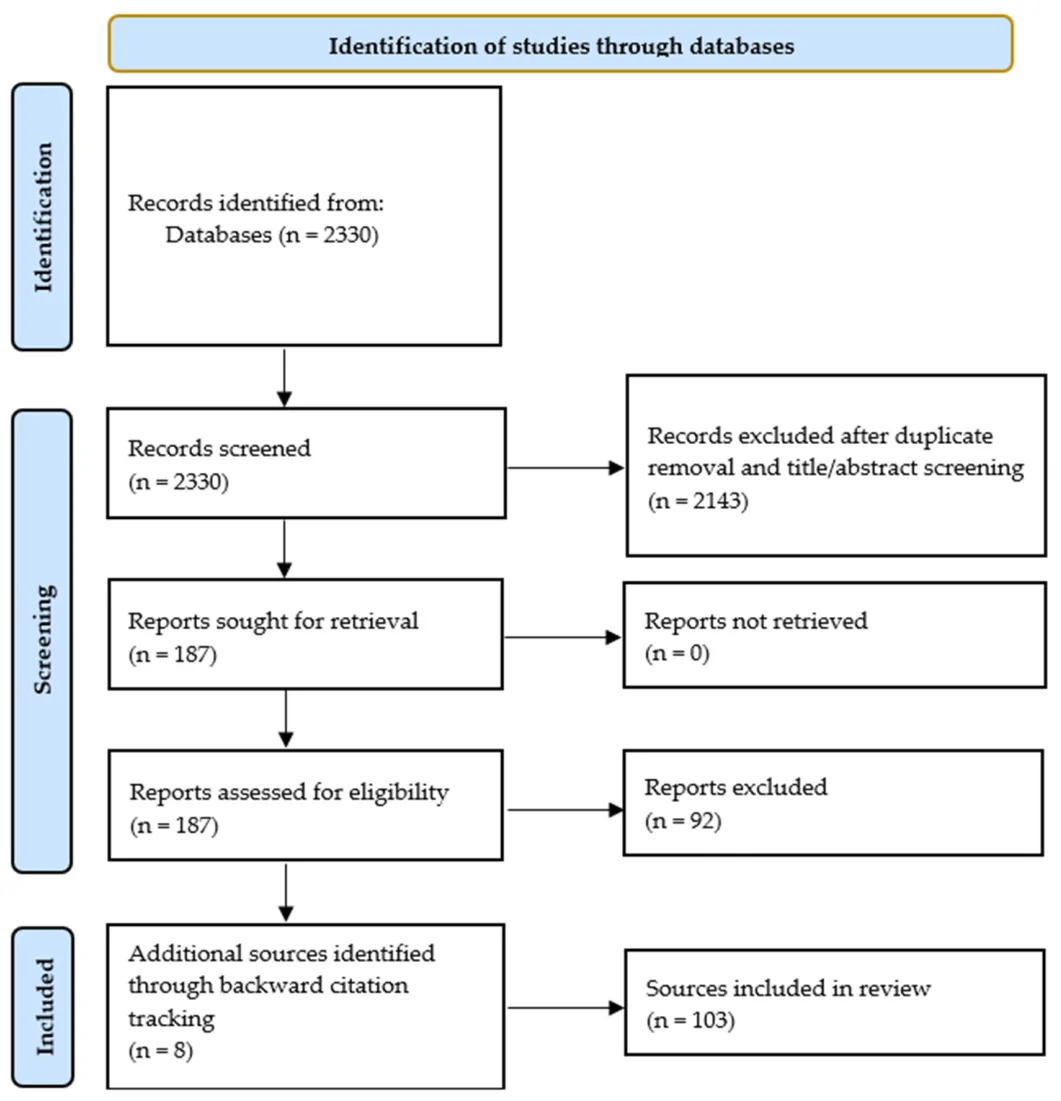
Flow diagram of the literature search and study selection process (single-reviewer screening), adapted from the PRISMA 2020 flow diagram [69].

The three queries returned a combined total of 2330 records. Duplicate removal and title/abstract screening were conducted as a single combined pass, consistent with the narrative-review approach declared above. Together, these processes resulted in 2143 records being excluded, leaving 187 records for full-text assessment. Of these, 92 were excluded on the basis of the criteria stated above, leaving 95 studies. A further eight sources, comprising regulatory and normative documents, a reference textbook on vehicle dynamics, and foundational methodological studies published before the start of the search window, were identified through backward citation tracking of the included papers and added to the corpus. The final corpus therefore comprises 103 sources, of which 79 (77%) were published in 2019 or later, reflecting the accelerated research activity driven by the commercial expansion of electric and autonomous vehicles. The complete selection process is summarized in Figure 1.

Beyond this search-derived corpus, four additional references [69,70,71,72] were incorporated during the revision process to support specific methodological points—the PRISMA 2020 reporting guidance, a functional-safety standard, an electromagnetic-compatibility reference, and a technology-readiness-level framework—bringing the total number of cited works to 107. These four references fall outside the structured search strategy described above and are identified as such at their respective points of citation.

### 1.3. Review Gap and Contribution of This Paper

Despite notable progress, the literature remains fragmented, with studies on wheel–road interaction rarely correlating with research on structural optimization and intelligent sensors. In this review, intelligent sensors are defined as sensing elements combined with embedded or associated processing capable of estimating a state or parameter not directly measurable by the sensor itself (criteria i–ii of the operational definition of the predictive tire presented above, consistent with the “sensor-equipped tire” category). This article aims to fill this gap with a comprehensive synthesis, integrating the phenomenology of wheel–road interaction, structural optimizations, and innovative compositions relevant to electric vehicles, intelligent sensors, and artificial intelligence (AI) methods for estimating TRFC and the forces generated during vehicle movement. These two parameters are emphasized because their ratio, the force currently transmitted relative to the maximum force available at the estimated friction coefficient, constitutes the instantaneous safety margin exploited by ABS and ESC control loops. This is illustrated by the vehicle-level connections established in Section 7, where friction coefficient and lateral force are estimated with RMSE values of at most 0.037 and 66 N, respectively [73]. Unlike slower-varying parameters such as wear or inflation pressure, which influence this margin indirectly, TRFC and force estimation provide the direct, real-time inputs required for safety-critical intervention.

The objective is to highlight the connection between these directions and the concept of the predictive tire, an optimized structure that not only transmits forces, but also provides essential information for predictive vehicle control, with direct applicability for electric and autonomous vehicles.

To address these issues, the paper is structured into the following sections: Section 2 describes wheel–road interaction and associated critical phenomena, Section 3 discusses structural optimizations and innovative materials, Section 4 analyzes smart sensors and estimation algorithms, and Section 5 studies methods for predicting the coefficient of friction. Section 6 integrates these directions into a common design framework for critical analysis, and Section 7 and Section 8 discuss the development prospects for electric and autonomous vehicles, as well as their limitations. Section 9 provides an integrated discussion of the results, highlighting cross-domain interdependencies and their implications for future tire technologies. The conclusions highlight the contribution of this synthesis and set out an agenda for the development of multifunctional tires for the mobility of the future.

## 2. Wheel–Road Interaction, Energy Losses and Contact Dynamics

Wheel–road interaction represents the fundamental interface through which forces are transmitted between the vehicle and the road surface, governing traction, stability, and energy efficiency. This interaction is characterized by complex nonlinear phenomena, including slip generation, contact patch deformation, and transient adhesion conditions, which are influenced by tire properties and external operating conditions [15,63,68,74,75,76].

Understanding these mechanisms is essential not only for improving vehicle dynamics, particularly in the context of electric and autonomous vehicles, where higher loads, torque delivery, and safety requirements impose strict constraints on tire performance, but also for calibrating the sensing architectures described in Section 4, since structural parameters constrain sensor placement, signal fidelity, and estimation accuracy.

### 2.1. Energy Losses from Slip and Control Methods

A critical aspect of wheel–road interaction is the energy loss associated with slip, which influences energy consumption, tire wear, and vehicle stability [14,77,78]. This phenomenon results from the continuous deformation of the tread elements and micro-sliding within the contact patch [79], becoming an important indicator of both traction efficiency and overall driving safety. Various control allocation strategies have been proposed to minimize tire-slip power losses in distributed drive configurations, from workload usage optimization [80] to model predictive approaches [78,81]. Recent work has extended this toward integrated slip-energy dissipation and stability coordination [14,57].

In [78], the classic Tire Workload Usage-Optimization (TWUO) method is compared with the advanced Model Predictive Control-Allocation (MPCA) method. The analysis shows that TWUO leads to unbalanced tire usage [78], by overloading the front axle. This induces an increase in longitudinal and lateral slip, which causes high energy losses and accelerated tire wear. In contrast, MPCA, due to its anticipatory nature, redistributes torque to individual axles and wheels, reducing energy losses by up to 50% in the longitudinal direction and by approximately 35% in the lateral direction.

The comparative effects of the two methods are summarized in Table 2, which highlights the advantages of the MPCA method in terms of directional stability and the autonomy of electric vehicles [78]. As indicated by the Validation level column in Table 2, these results derive exclusively from numerical simulation (CarSim-based). No experimental validation of these percentages against real vehicle data is reported in the source study.

### 2.2. Contact Patch Dynamics and Aquaplaning Critical Parameters

The size and shape of the contact patch are fundamental parameters of wheel–road interaction, determining the ability to transmit longitudinal and lateral forces. Studies [34,82,83] have demonstrated that there is an almost linear relationship between vertical load and contact patch length; increasing the load from 500 N to 6500 N extends the length of the tire–road contact surface from 0.03 m to 0.10 m. Although this increase favors the transmission of longitudinal forces, it can lead to uneven pressure distribution over the contact area and reduced vertical stiffness, affecting cornering stability.

On wet surfaces, behavior is much more critical, as increased speed causes a rapid reduction in the contact patch area, to the point where the tire almost completely loses contact with the road surface. In the wet-track tests of [34], the contact length of a new tire began to decrease above about 40 km/h, and full aquaplaning occurred at approximately 90 km/h at 2.2 bar (95 km/h at 2.6 bar). For a worn tire, the critical speed was approximately 75 km/h. Increasing the inflation pressure delays the moment of contact loss, but does not eliminate it, and tread wear accentuates this effect.

Aquaplaning is one of the most dangerous situations in wheel–road interaction [28], as the loss of contact between the tire and the road surface leads to the almost total disappearance of traction or braking forces, but also to the loss of the vehicle’s ability to move in the direction commanded by the driver [28,30,84]. The standardized procedure of the German Association of the Automotive Industry (VDA) describes the critical aquaplaning speed as the value at which the longitudinal tire slip reaches approximately 15% at the end of the wet section [29,85].

Experimental analysis [29] shows that this threshold is strongly influenced by four main parameters, namely inflation pressure, vertical load, water depth, and tire type. According to the results in [29], increasing the pressure from 2.2 bar to 2.6 bar delays the development of aquaplaning due to the reduced volume of water retained in the contact patch. However, the benefit is diminished in the case of worn tires, where the drainage grooves have reduced water evacuation capacity. In addition, the high vertical load causes higher pressure in the footprint, which temporarily increases stability but accelerates the formation of the water film at high speeds.

A summary of these effects is shown in Table 3, where it should be noted that the parameters mentioned (inflation pressure, vertical load, water depth, and tire type) may have contradictory effects: they can delay aquaplaning on wet roads or, under certain pressure conditions, accelerate the loss of contact on wet and dry roads.

These results highlight that aquaplaning cannot be completely prevented by increasing pressure, but only delayed, and wear and tear may cancel the benefits achieved. For electric vehicles [13,14], the additional weight and different distribution of load increase sensitivity to this phenomenon, which requires the development of prediction and adaptive control algorithms.

## 3. Structural Optimization of Tires

Structural optimization of tires is a key focus in modern automotive engineering, with direct implications for NVH, stability, and particularly the energy efficiency of electric vehicles. Given that electric vehicles are heavier than conventional vehicles, structural irregularities and material characteristics become critical factors for comfort and driving range [10].

Beyond traditional design approaches, tire optimization is evolving toward multi-objective frameworks that must simultaneously balance conflicting requirements, such as high grip, low rolling resistance, durability, and reduced energy losses. In this context, radial stiffness, carcass deformation, and contact pressure distribution play a critical role in defining both dynamic performance and energy efficiency, while also establishing the mechanical boundary conditions within which embedded sensors operate, making their optimization a prerequisite for reliable intelligent tire sensing.

Furthermore, multi-physics modeling and innovative composite materials enable a deeper understanding and control of tire behavior under real operational conditions, particularly for electric vehicles with high torque demands and dynamic load variations.

### 3.1. Radial Force Variation and NVH Impact

Radial force variation (RFV) is a determining factor of additional excitations relative to the contact patch, generating fluctuations in longitudinal and vertical forces and amplifying the vibrations transmitted to the vehicle structure. Experimental and numerical studies show that the vibration and noise transmitted to the vehicle occupants, from road irregularities and from the powertrain, degrade NVH comfort [10,48,86,87], and that uneven tire radial stiffness further amplifies the effect of road irregularities on seat vibration [87]. From a practical point of view [87], an RFV not exceeding about 50 N is assumed to have no negative effect on driving comfort. Values above about 75 N may indicate hidden material defects affecting radial stiffness, and an RFV of around 80 N can already cause a noticeable deterioration of comfort on a good road surface (approximate values, which may be up to 50% higher for larger vehicles). In the source study [87], the dynamic load coefficient (DLC), an independent measure of driving safety distinct from vibration-based comfort assessment, remained below the warning value usually assumed for it (0.3) across the tested speed range, even where vibration comfort limits were exceeded. This indicates that comfort degradation does not necessarily coincide with a loss of driving safety margin.

### 3.2. Multi-Objective Optimization Using Genetic Algorithms and Neural Networks

While genetic algorithm-based structural optimization is primarily a mechanical engineering topic, its relevance to intelligent tire sensing is direct and fundamental: carcass geometry and stiffness distribution define the boundary conditions within which sensors operate, making structural optimization a prerequisite for accurate signal interpretation.

Structural optimization using genetic algorithms and neural networks [88] aimed to reduce the peak strain energy density at the belt edge and the peak shear strain of the carcass. The design variables were the carcass path, the belt width, the angle of the two middle belt layers, and the rubber modulus of the base strip and sidewall. The predicted reductions were 21% and 39%, respectively, compared to the initial design (Table 4). Direct finite element analysis of the optimized design gave reductions of about 20% and 34%. These results indicate improved durability, as characterized by these two indicators, but the method remains limited by its high complexity and an approximation error of about 7% for the peak shear strain relative to FEM (Finite-Element Method) analysis, limitations that necessitate hybrid or simplified solutions for industrial implementation. Similar structural analysis approaches have been applied to related loading scenarios, including digital twin frameworks for tire–rim interaction [76] and radial-impact validation [89].

### 3.3. Advanced Multi-Physics Modeling (ST-SI-TC-IGA)

The ST-SI-TC-IGA framework is presented here not as a sensing technology but as a modeling tool that enables high-fidelity simulation of the stress and deformation fields within which tire sensors must function, providing a computational reference against which sensor calibration models can be validated.

Advanced multi-physics tire modeling has evolved from coupled FEM-DEM (Finite-Element Method/Discrete-Element Method) approaches [68] and digital-twin frameworks [76] toward unified space-time formulations [90]. 

The framework ST-SI-TC-IGA [90] combines tire deformation, contact with the road surface, and aerodynamics in a single space-time computational framework, in contrast to separate FEM and CFD (Computational Fluid Dynamics) analyses. Its key components are the ST (Space–Time) Variational Multiscale method; the SI (Slip-Interface) method, which handles the rotation of the tire relative to the surrounding mesh; the TC (Topology-Change) method, which handles the topology change created by tire–road contact; and IGA (Isogeometric Analysis), which offers high numerical accuracy through the use of CAD (Computer-Aided Design) splines.

The ST-SI-TC-IGA framework [90] enables high-fidelity computational analysis of tire aerodynamics with near-actual geometry, road contact, and tire deformation, although the study does not report quantified accuracy metrics. For fluid films between the tire and the road, the authors report solution quality and computational cost comparable to a Reynolds-equation model. In the context of electric vehicles, the coupling between deformation and aerodynamics may be relevant for reducing rolling resistance and modeling structural noise, which [90] does not address directly.

### 3.4. Innovative Composite Materials for Electric Vehicles

Composite material selection directly influences sensor integration feasibility; rubber matrix viscoelasticity determines signal-to-noise ratios in PVDF and FBG systems; and thermal properties affect long-term sensor stability, making material characterization inseparable from intelligent tire design.

Advanced composite materials represent a major optimization direction [17,31,46,47,91,92]. As shown in Table 5 [13], among the compared materials, Treated Distillate Aromatic Extract (TDAE), R131, High-Temperature Processed Material (HT), and Silane-Terminated Telechelic Polybutadiene (STPB), only STPB combines high mechanical strength (~19 MPa), the highest wear resistance (~225 mg mass loss), and a low loss factor at 60 °C, indicating reduced rolling resistance and, potentially, an improved range. These results support the relevance of open silica network-based composites for next-generation tires.

Comparative results [13] show that only STPB manages to combine silica micro-dispersion with increased network density, lowering the loss factor at 60 °C by about 14 ÷ 16% and the abrasion loss by about 10 ÷ 11% compared to the TDAE reference. TDAE and R131 offer acceptable stability but do not bring major improvements in efficiency, while HT shows the lowest loss factor at 0 °C (a wet-grip indicator) and the highest loss factor in the strain sweep, which is unfavorable for the range of electric vehicles.

Thus, STPB appears to be a reference material for electric-vehicle tires, offering a compromise between safety, efficiency, and durability, although its sensitivity to temperature and humidity variations requires further research to validate its performance under real operating conditions [13]. This synthesis focuses on rubber compound formulation. The steel belt and cord reinforcement, though structurally essential to radial tire construction, is considered in this review only through the structural optimization of the belt layers in [88] (Section 3.2); its role as a composite material, and its interaction with sensor integration, are not analyzed here and would complement the present synthesis.

## 4. Smart Tires and Integrated Sensors

Sensory integration at the tire level transforms the contact patch from a purely mechanical interface into a primary source of real-time data for vehicle dynamics and control. By embedding advanced sensing technologies directly into the tire structure, it becomes possible to monitor critical parameters such as deformation, contact forces, slip conditions, and friction coefficient with high accuracy.

This transformation enables a shift from indirect estimation methods toward direct measurement of wheel–road interaction, potentially improving the performance of vehicle control systems such as ABS, ESC, and torque vectoring, as well as advanced driver assistance systems (ADAS). Moreover, the tire evolves into a distributed sensing platform, capable of complementing traditional perception systems (camera, radar, LiDAR), particularly in scenarios where external sensing is limited.

### 4.1. Distributed Strain Sensors

**Figure 2:**
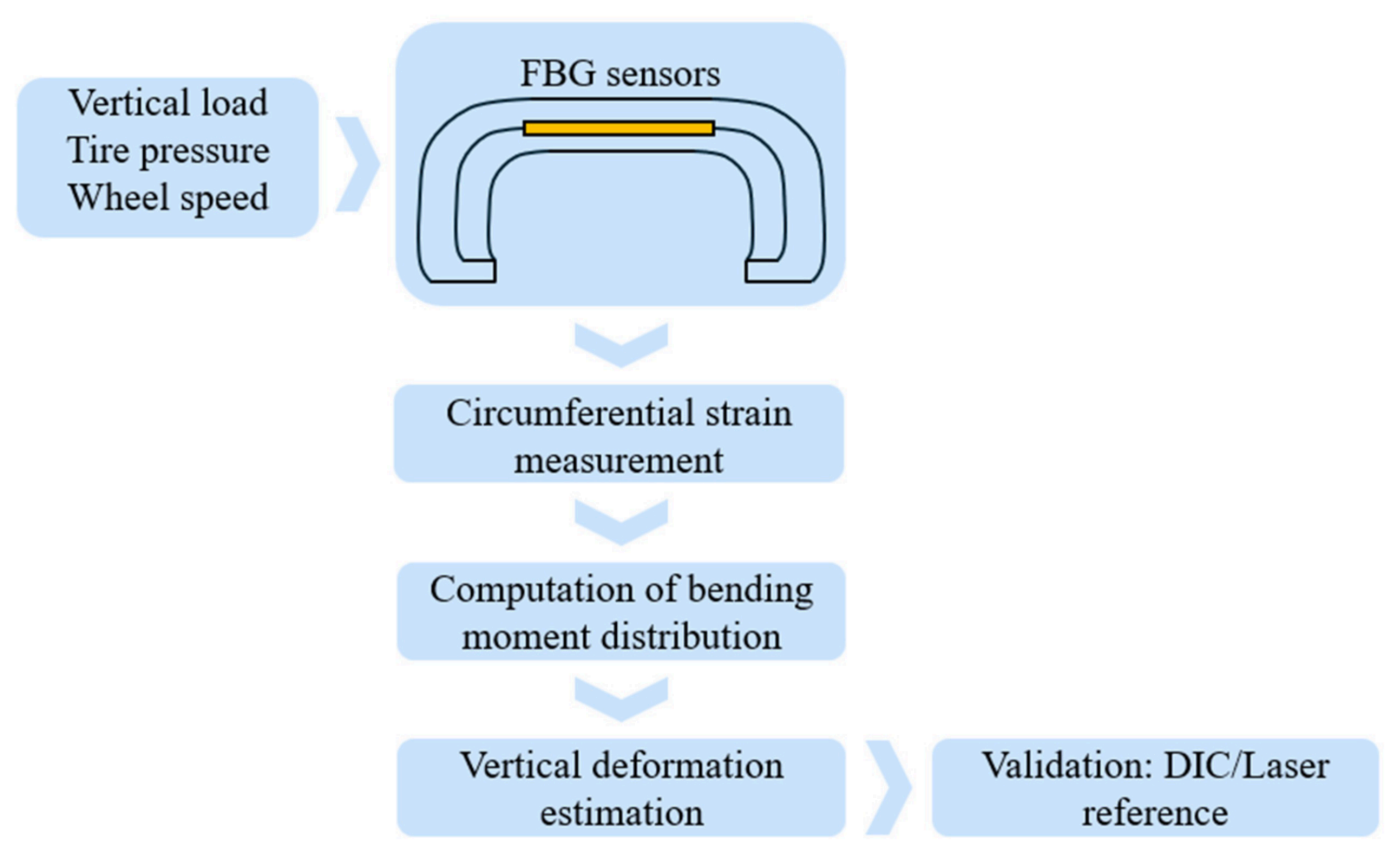
Methodological diagram of continuous tire deformation measurement using long-gauge FBG sensors, based on [93]. The orange line indicates the optical fiber containing the Bragg grating; blue boxes represent the processing steps.

The integration of long-gauge Fiber Bragg Grating (FBG) distributed strain sensors into the tire structure (Figure 2) enabled continuous and direct measurement of circumferential stress variations, providing essential data on wheel behavior in contact with the road [93]. In laboratory tests [93] under different loads and pressures, the deformation calculated from the strain signals agreed with optical reference measurements, with a maximum deviation of 3.8% from digital-image correlation (DIC) in static tests and good agreement with a laser displacement sensor on a rotating tire, supporting the robustness of the methodology under the tested conditions. This approach complements strain-based grip identification methods [39] and severe-maneuver loss-of-grip characterization [42], both relying on related deformation-sensing principles [36].

However, there remains a trade-off between sensitivity and reliability, as shortening the measurement length increases local sensitivity [93], but amplifies noise and reduces long-term durability, whereas longer lengths filter out variations and provide better overall accuracy at the expense of resolution.

In the context of electric and autonomous vehicles, where real-time estimation of contact parameters is essential, these sensors represent a promising solution but require configuration optimization to maintain an optimal cost–performance ratio [93].

### 4.2. Estimating the Vertical Reaction Force of the Road with Respect to the Wheel and the Mass of the Vehicle Through Artificial Intelligence and Fuzzy Logic

**Figure 3:**
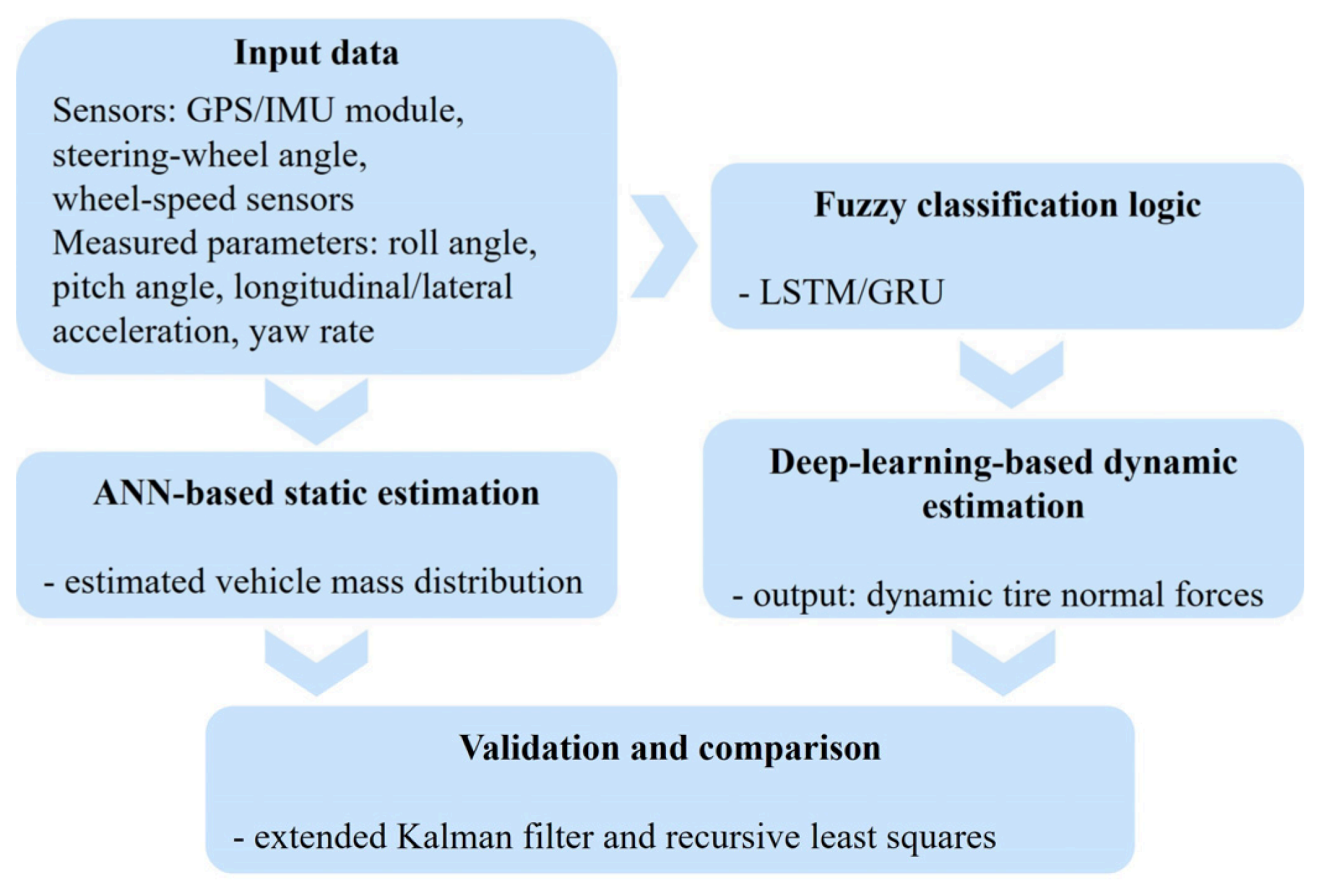
Framework for tire normal force and vehicle mass estimation using AI and fuzzy logic, based on [8].

Estimating the normal force exerted on the tire and the mass of the vehicle remains essential for dynamic control and calibration of ABS/ESC systems. The algorithm proposed in [8] (Figure 3), based on neural networks and fuzzy classifiers integrated with a 9-DOF hardware–software model, has been reported to achieve both high accuracy (Table 6) and robust adaptability to variable road conditions, outperforming Extended Kalman Filters (EKF) and Recursive Least Squares (RLS) in the reported tests, which suggests its applicability to conventional and electric vehicles. This complements smart-tire-based vertical force estimation algorithms [94] and classical sideslip/cornering stiffness observers [38].

### 4.3. Accelerometers Integrated into the Tire

**Figure 4:**
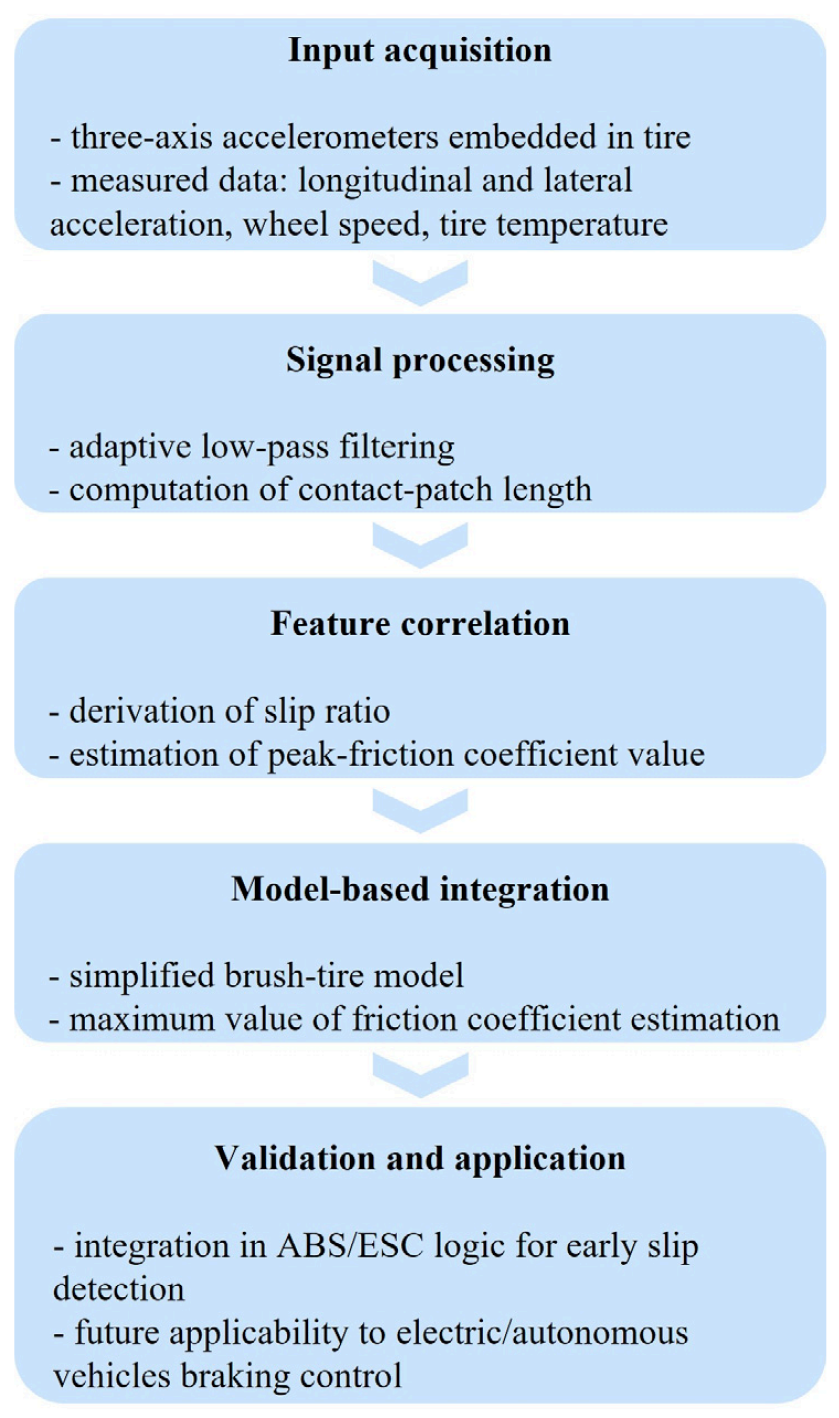
Framework for tire longitudinal dynamics estimation, based on [95].

Accelerometers mounted in the tire structure have been shown to be effective tools for identifying the transition between the adhesion and slip regimes (Figure 4), offering a potential advantage over indirect estimates. The results summarized in Table 7 indicate that, in the braking maneuver analyzed in [95], small vibrations indicating the onset of sliding were already detectable in the contact patch at a brake slip of about 3.7%, whereas full sliding was observed at 13.7%.

Compared to flexible embedded force sensors [82], accelerometers offer simpler integration but require careful calibration against geometric effects such as toe angle variation, consistent with broader findings on tire-sensor applications [36].

In addition [95], a toe angle of ~1.5° produced measurable vibrations already at small slips, as a consequence of the resulting lateral slip, which shows the sensitivity of accelerometers to wheel alignment. This approach could support not only early detection of loss of traction, but also the optimization of ABS and ESC, although large-scale validation requires extending the studies to a larger number of tires and varied road conditions.

### 4.4. Deformation Sensors Integrated into the Tire Casing

**Figure 5:**
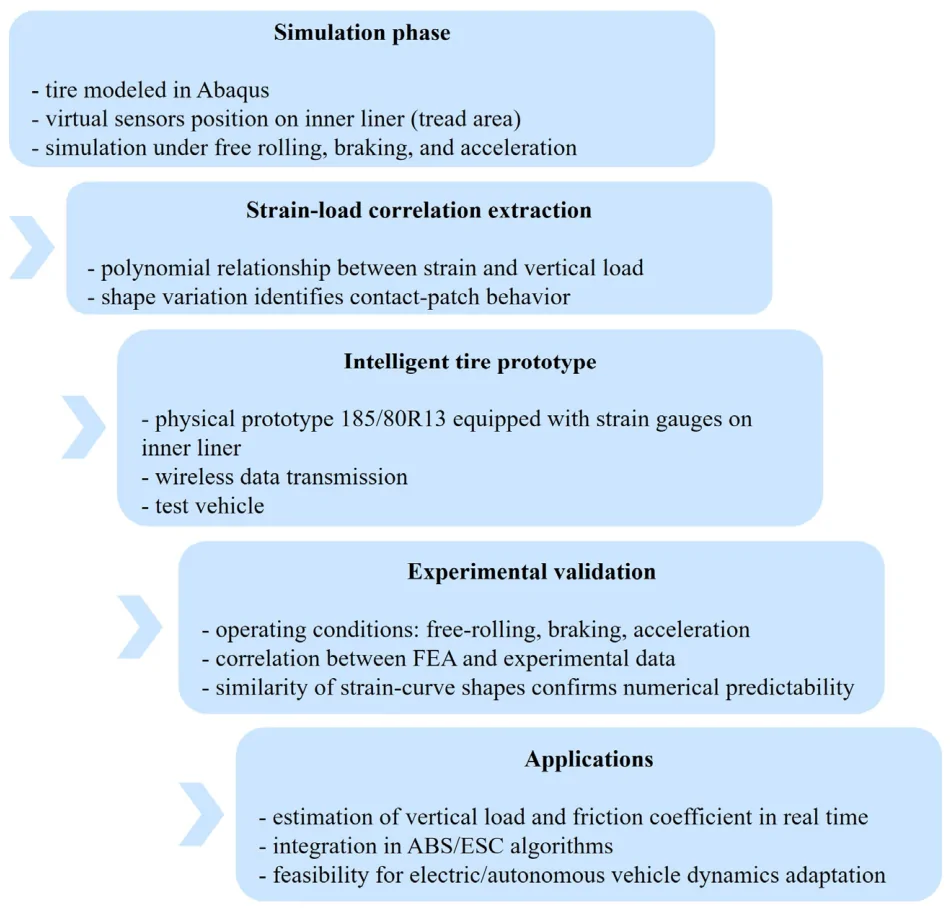
Framework for intelligent tire strain-based estimation, based on [96].

Strain-gauge sensors incorporated directly into the casing layers (Figure 5) have been used to distinguish dry from snow-covered pavement through analysis of strain variations in the casing [96]. These results align with strain-based loss-of-grip characterization under severe maneuvers [42] and earlier theoretical work on grip identification from strain information [39].

Experimental validation [96] confirmed the qualitative trends of the numerical predictions, although the simulated strain amplitudes were up to five times higher than those measured, which reinforces the use of these data in wheel–road contact model calibrations and friction coefficient estimation.

The detailed results are summarized in Table 8, which highlights that the measurements addressed can be used as indirect indicators for normal load, transition to slippage, and variable adhesion conditions. The advantages of the method are its simplicity of integration and lower costs compared to solutions based on three-dimensional accelerometers or piezoelectric sensors, but practical application requires careful calibration and compensation for thermal effects [96].

### 4.5. PVDF Piezoelectric Sensors for Slip-Angle Estimation

**Figure 6:**
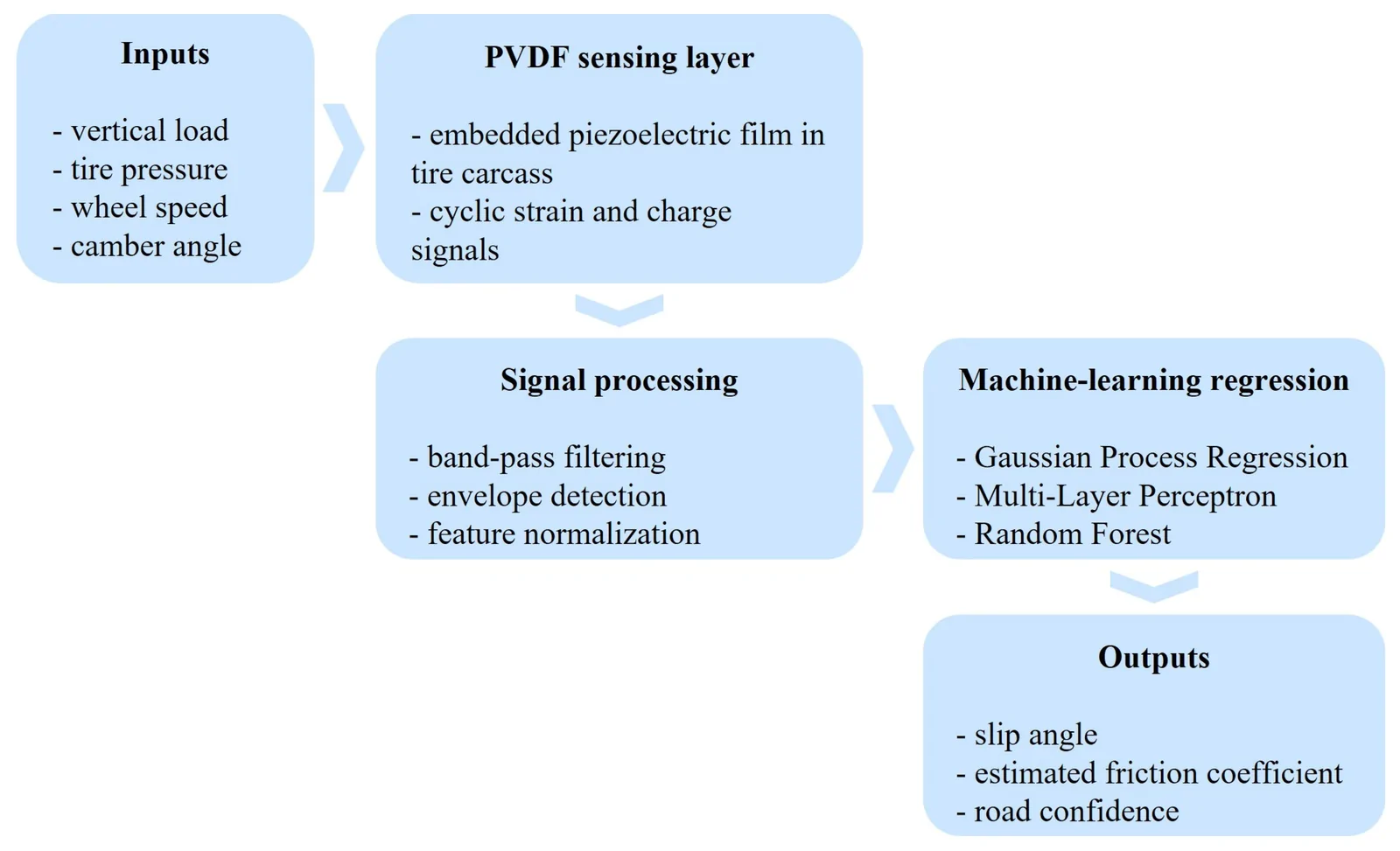
Simplified framework for slip-angle estimation using PVDF tire sensors, based on [97].

Piezoelectric sensors based on polyvinylidene fluoride (PVDF) film are an innovative solution for directly estimating the tire-slip angle (Figure 6), an essential parameter for handling and optimal ABS and ESC operation. Mounted in the layers of the casing, these sensors capture the cyclic deformations generated during rolling, and the signals obtained are directly correlated with the variations in longitudinal and lateral forces [97]. The same sensing technology has also been applied to direct friction coefficient estimation [37], indicating the versatility of PVDF-based intelligent tire architectures across multiple estimation tasks, consistent with broader tire sensor applications [36].

The results summarized in Table 9 highlight the influence of factors such as variable load, inflation pressure, vehicle speed, and degree of wear on the amplitude of PVDF signals, indicating the potential of these sensors as indicators of available grip.

Furthermore, machine-learning algorithms, such as Gaussian Process Regression (GPR), Multi-Layer Perceptron (MLP), and Random Forest (RF) [97], have been applied to slip-angle estimation, with GPR giving the lowest errors (approximately 5 ÷ 7%).

In particular, the GPR model achieved the best performance [97], with a mean absolute percentage error (MAPE) of 5.5% in cross-validation and 7.2% in extreme maneuvering scenarios (wet road, high slip angles), which the authors regard as an acceptable range.

The PVDF solution [97] stands out among intelligent-tire sensor types for its low cost, compact size, and lack of need for its own power supply beyond the signal transmission module, which gives it solid prospects for integration into smart tires designed for electric and autonomous vehicles.

### 4.6. Embedded Force Sensors and Three-Dimensional Force Measurement

**Figure 7:**
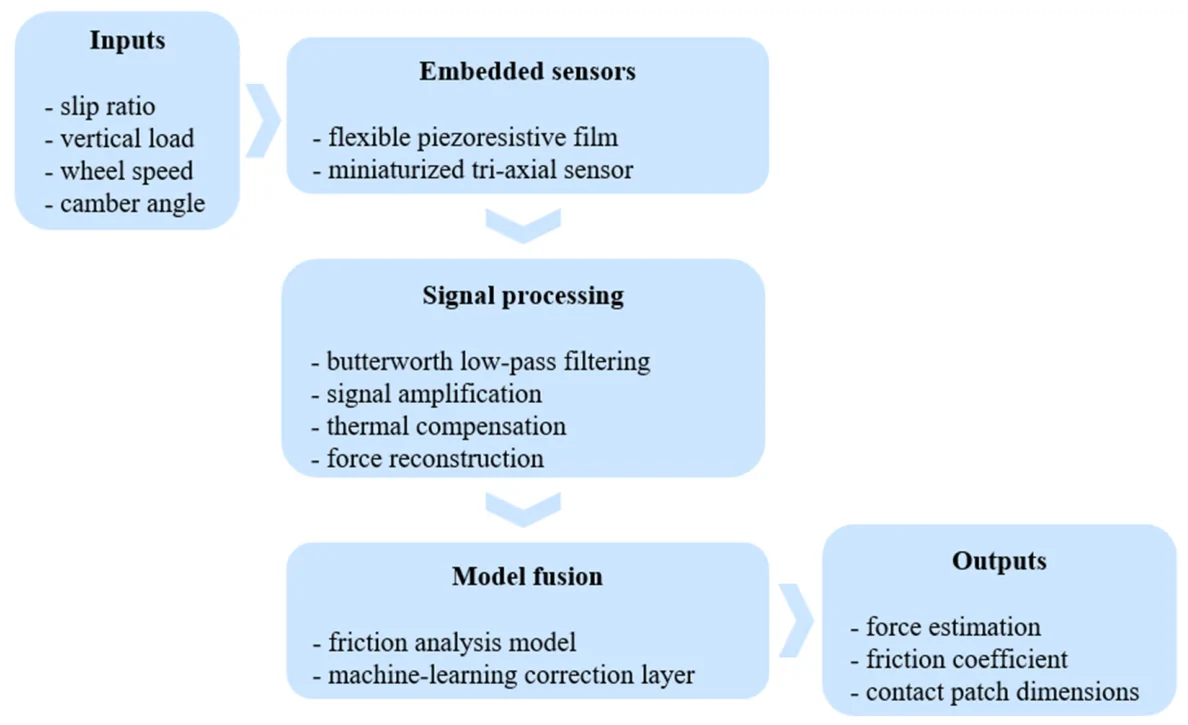
Simplified framework for triaxial force estimation in intelligent tires, based on [32,82].

The strain-gauge sensors discussed in Section 4.4 provide indirect indicators of load and adhesion conditions, and the PVDF sensor discussed in Section 4.5 measures deformation, from which specific quantities such as load, adhesion conditions, or slip angle are inferred through models. An emerging research direction is the development of embedded force sensors that measure forces directly. Recent approaches include flexible piezoresistive force-sensing films embedded in the tread rubber, which measure the vertical and longitudinal force components and, combined with a physical tread model, allow the friction force to be estimated in real time [82]. Another approach is a miniaturized triaxial force sensor bolted to the belt beneath a tread block, which measures the three force components F_x_, F_y_, and F_z_ on a single tread block, and was tested on a flat-belt test bench (Figure 7) [32].

The results summarized in Table 10, obtained with the triaxial sensor of [32] on a flat-belt test bench, show how each force component of a single tread block responds to vertical load and slip angle. The main advantage of the flexible film of [82] lies in its mechanical flexibility and compatibility with tire deformation. The limitations reported in the sources include the need to calibrate the triaxial sensor against belt deformation and the local drilling of the belt required to mount it [32]. For the flexible film, they include the sensitivity of the signal to the contact between film and rubber, the lack of lateral-force measurement, and testing at low speed and load [82].

### 4.7. h-TPMS Systems for Detecting Tire Wear and Road-Surface Conditions

Tire-pressure monitoring systems are the most widely mandated intelligent tire technology for series-production vehicles: UNECE Regulation No. 64 [98] defines the technical requirements for TPMS, whose fitment became mandatory in the European Union for new M1 passenger vehicle types from November 2012 and for all new M1 vehicles from November 2014.

The mandate was subsequently extended in the European Union to the M2, M3, N1, N2, N3, O3, and O4 vehicle categories, covering buses, vans, and heavy commercial vehicles, with obligations entering into force in July 2022 for new type approvals and in July 2024 for all new registrations, based on the technical requirements of UNECE Regulation No. 141 [99]. This significantly expands the addressable context for h-TPMS architectures.

**Figure 8:**
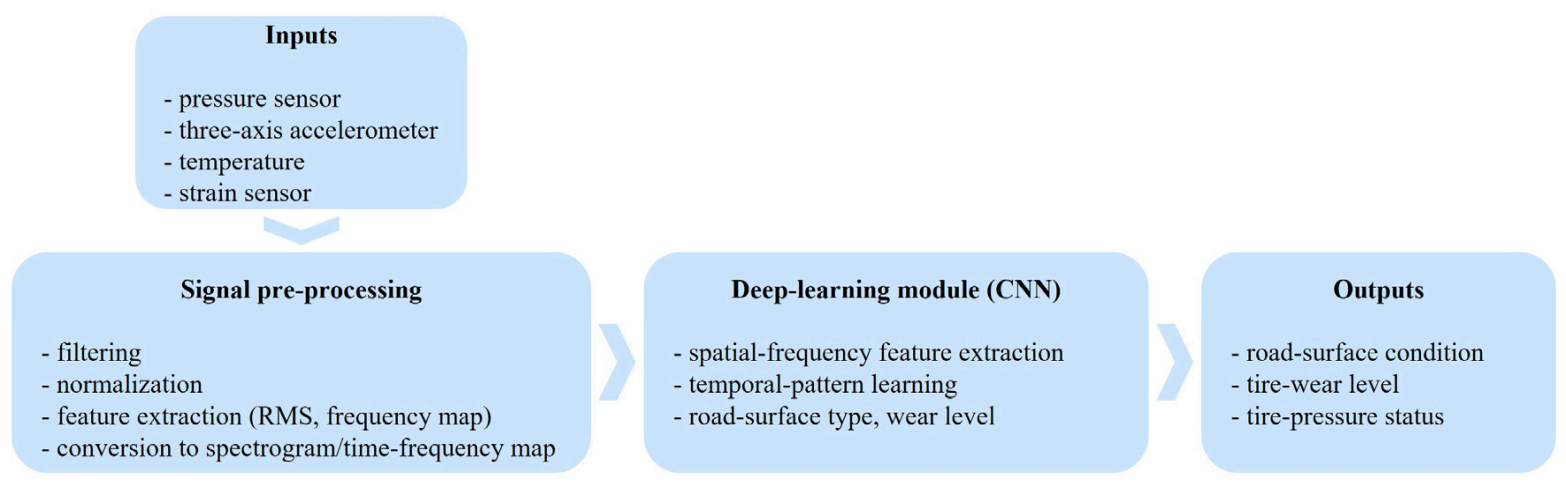
Hybrid TPMS architecture for road and tire condition recognition using CNN, based on [59,100,101].

The integration of Convolutional Neural Network (CNN) algorithms has been reported to enable more accurate classification of road-surface type and wear than conventional methods based on statistical thresholds (Figure 8). According to Table 11, CNN-based systems are reported to remain stable under varying pressure, load, and wear, and to outperform LSTM, RF, and SVM classifiers, supporting their applicability for electric and autonomous vehicles [59,100,101].

Thus, hybrid TPMS (h-TPMS) [100] evolves beyond pressure monitoring, with the underlying index shown to reliably detect a 20% pressure loss despite parameter uncertainty, and with the potential for direct integration into the vehicle’s electronic control unit (ECU) [102].

## 5. Prediction of the Friction Coefficient

Within the emerging paradigm of the predictive tire, the estimation of the TRFC becomes a central functionality, enabling the tire to act as an active source of information for vehicle dynamics and control. As TRFC directly defines the maximum transferable forces at the contact interface, its accurate real-time estimation is essential for ensuring stability, safety, and energy efficiency, particularly in electric and autonomous vehicles operating under highly variable road conditions [18,19].

Unlike conventional approaches based on conservative assumptions or indirect indicators, predictive tire architectures continuously infer available friction by integrating vehicle dynamics, sensing technologies, and data processing. This shift from passive to active prediction supports ABS, ESC, torque vectoring, and ADAS in operating closer to the physical limits of adhesion [62,73].

### 5.1. Estimating the Friction Coefficient Using Vehicle Signals, Without Sensors Mounted Inside the Tire

**Figure 9:**
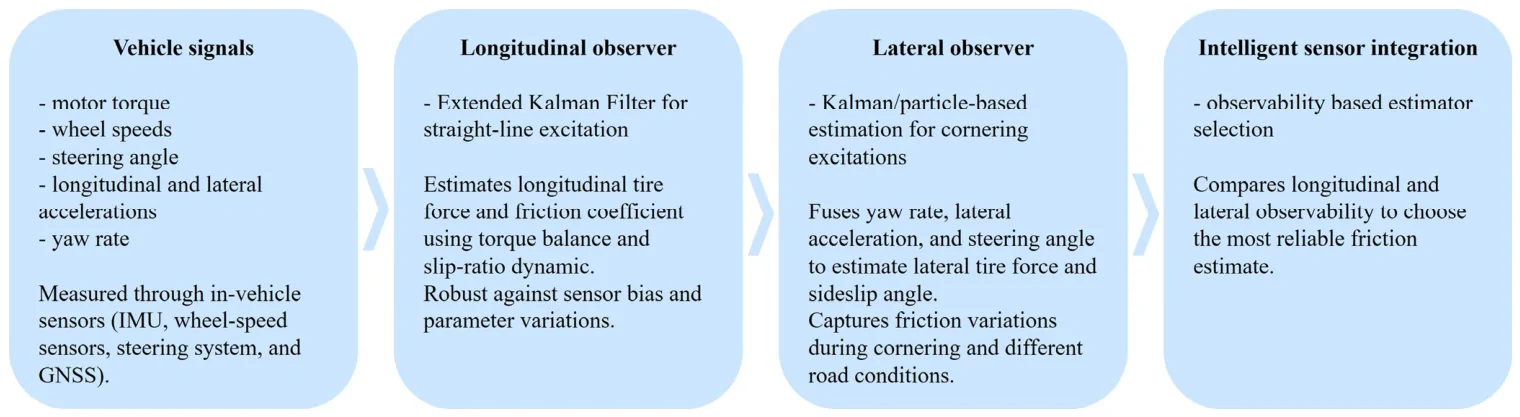
Tire–road friction coefficient estimation concept applied in 4WID/4WIS electric vehicles, based on [73].

The approach proposed by [73] uses a particle filter (PF)-based tire–road friction estimation method for four-in-wheel-motor-drive electric vehicles (FIWMD EVs). Unlike solutions based on integrated sensors, the method captures only the dynamic variables of the vehicle, such as angular velocities, motor torques, longitudinal and lateral accelerations (Figure 9), thus eliminating additional costs.

Experimental results [73], obtained on split-μ scenarios (different friction coefficients under left and right wheels) and sinusoidal steering maneuvers, show an RMSE of 0.037 for the proposed PF-based estimator, compared to RMSE 0.061 for an extended Kalman filter (EKF) baseline. Individual wheel RMSE values ranged from 0.008 to 0.037 across braking, acceleration, and steering test scenarios (Table 12). Longitudinal tire forces were estimated with RMSE values between 55.7 N and 144.7 N at wheel level, lateral tire forces between 42.6 N and 58.0 N at wheel level (58.9 N and 66.0 N at axle level), and the vehicle sideslip angle with an RMSE of 0.077°. The response time of the proposed PF estimator was 535 ms, compared to 567 ms for the EKF baseline, with the estimator additionally validated on a processor equivalent to mainstream production vehicle control units, indicating hardware compatibility beyond research-grade computing platforms.

This sensor-free philosophy is shared by moving optimal estimation strategies [58] and hierarchical estimation frameworks [23,54], all developed for similar four-wheel-motor-drive EV architectures, as well as cascade-observer structures robust to varying road conditions [64].

### 5.2. Data-Driven Framework with Uncertainty Assessment

**Figure 10:**
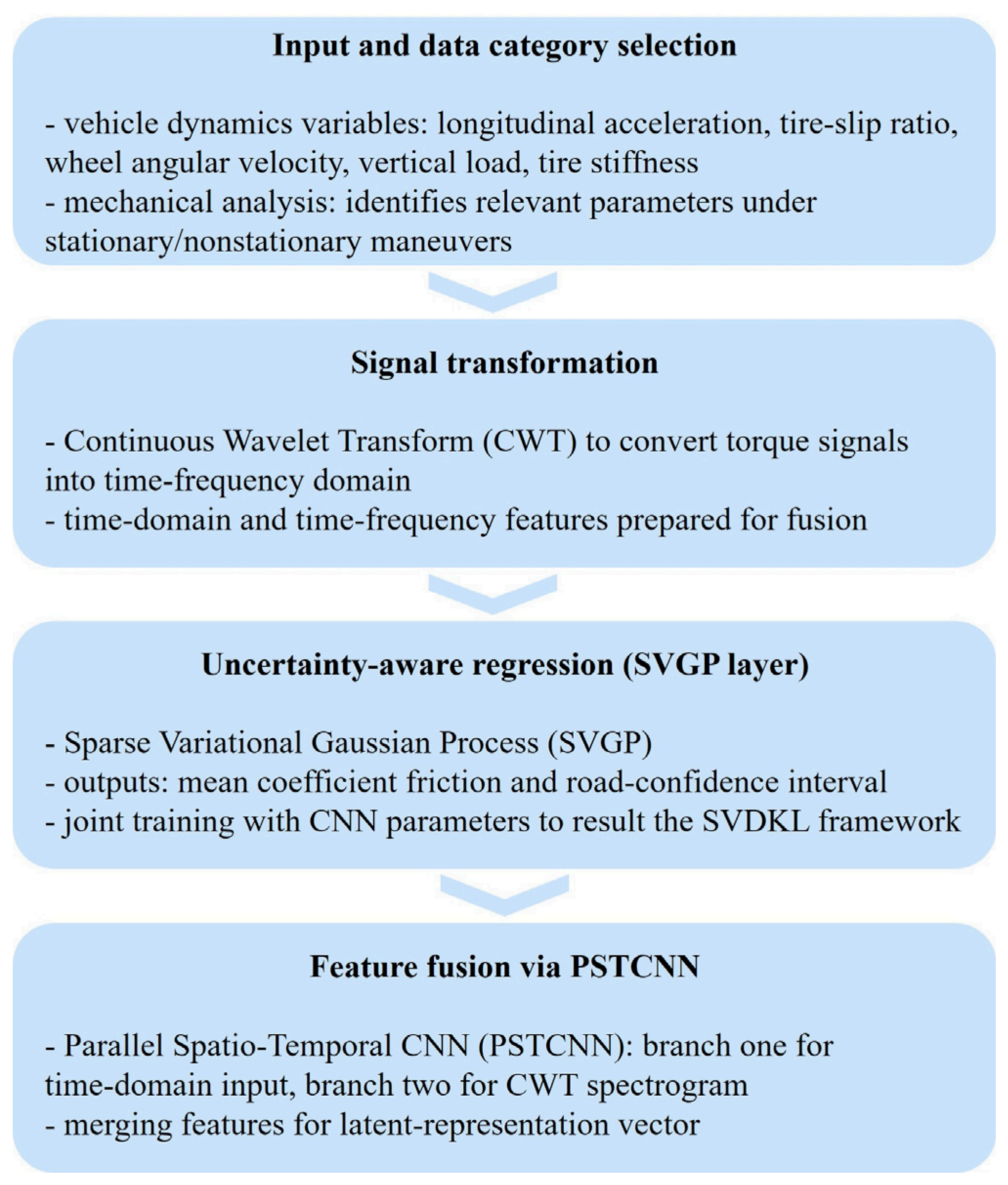
Data-driven tire–road friction estimation using SVDKL in electric vehicles, based on [33].

The paper [33] proposes a data-driven framework for estimating the TRFC for electric vehicles with motors integrated in the wheel hub. The approach is based on the selection of relevant data categories through mechanical analysis of vehicle dynamics (for stationary and non-stationary maneuvers), avoiding redundancy of inputs in neural networks. Next, multi-domain characteristics (time and time-frequency) are merged using continuous wavelet transform (CWT) for motor torque signals. Finally, a stochastic variational deep-kernel learning (SVDKL) framework is used, which integrates a parallel spatial-temporal convolutional neural network (PSTCNN) and a sparse variational Gaussian process (SVGP), allowing both the mapping of spatiotemporal features and the evaluation of uncertainty (Figure 10).

The proposed method outperforms classical techniques, including moving horizon model-driven estimators [58], and is particularly advantageous over hybrid image-dynamics fusion approaches [103], which require additional visual-sensing infrastructure.

The results from simulations and experiments [33] indicate that the proposed method outperforms classical techniques (slip-slope and conventional data-driven methods), especially under low excitation conditions (low slip ratio). In the simulated cases, the mean absolute errors (MAEs) were reduced by about 38 ÷ 83% relative to the methods compared, and the real TRFC values lay predominantly within 95% confidence intervals, supporting the uncertainty assessment.

The conclusion is that this combination of theoretical analysis of dynamic variables, multi-domain feature extraction, and integration of a probabilistic framework represents a promising direction for reliable and interpretable estimation of tire–road interaction in electric vehicles with wheel-integrated motors.

### 5.3. Combined Effect of Camber Angle, Slip Angle, and Wheel Load

**Figure 11:**
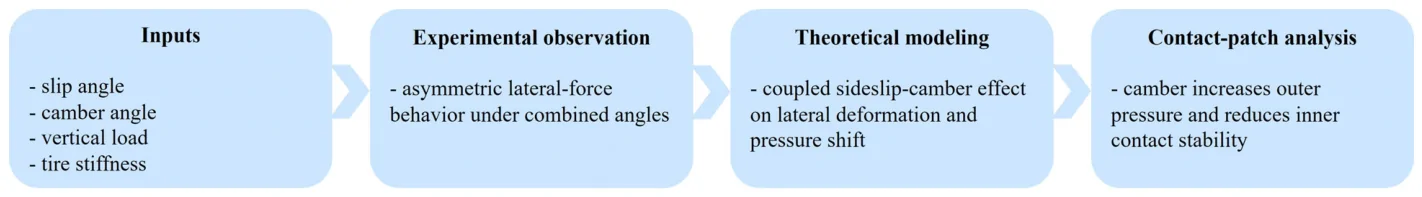
Tire–road friction coefficient estimation under combined sideslip and camber conditions, based on [66].

Estimating the tire–road friction coefficient (TRFC) becomes much more complex when the slip angle is combined with the camber angle, as the latter changes the pressure distribution inside the tire and the shape of the contact patch. In ref. [66], it was shown that, at high wheel loads, slip and camber angles of the same sign tend to increase the peak friction coefficient, while opposite signs tend to reduce it, generating an asymmetry in the tire’s behavior at the limit (Figure 11).

Discrete tire models developed for limit handling evaluation [53] provide theoretical context for this nonlinear interaction, which requires lateral-force validation not yet shown using the sensor [82].

The results reported in [66] and summarized in Table 13 show that, for the tires tested, the peak friction coefficient can be up to about 17% larger when the camber angle and slip angle have the same sign, and up to about 8% smaller when their signs are opposite, relative to pure sideslip. The effect is stronger at higher speed.

These variations become critical for electric vehicles [66], where higher masses and low-rolling-resistance tires significantly amplify the effect of wheel load redistribution on wheel–road contact. Therefore, the integration of the camber angle into friction coefficient estimation schemes can no longer be treated as simple corrections, but as an essential part of predictive models and dynamic-control algorithms.

### 5.4. Hybrid Estimation of the Friction Coefficient Using Kalman and Adaptive Fuzzy Networks

**Figure 12:**
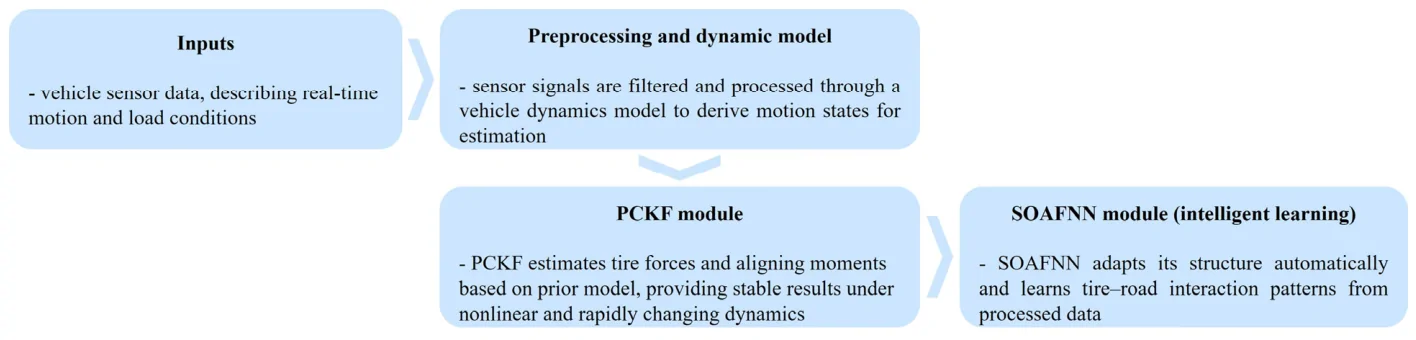
Combined model-based and intelligent estimation approach for robust, real-time TRFC prediction, based on [62].

In the case of electric vehicles with independent traction and steering on each wheel (4WID/4WIS), where dynamic behavior is highly nonlinear and dependent on rapid load variations, the authors of [62] propose a hybrid method that combines a prior knowledge-based Cubature Kalman Filter (PCKF) with a self-organizing asymmetric fuzzy neural network (SOAFNN). PCKF is used to accurately estimate longitudinal forces and self-aligning torque, while the SOAFNN replaces traditional methods by learning the actual characteristics of the tire through an asymmetric membership mechanism with reduced parameters (Figure 12).

This performance is comparable to that of IMM designs based on tire force observation [43], suggesting convergent advances toward hybrid model-data estimation architectures for 4WID/4WIS platforms, building on earlier corner-based robust estimation work [64].

Simulation results [62] show that the PCKF reduces the root mean square error (RMSE) of the longitudinal force by 26.6% and that of the self-aligning torque by 33.2% compared to the conventional CKF. In the experiments, the friction coefficient RMSE was about 20% lower than that of the RLS scheme, and the proposed scheme showed the fastest convergence when the coefficient changed.

Therefore, the integration between PCKF and adaptive fuzzy networks combines the robustness of model-based observers with the adaptability of artificial intelligence, making this method a promising candidate for further development toward ECU integration in advanced electric vehicles [62].

### 5.5. Transformer Networks for Estimating the Friction Coefficient

**Figure 13:**
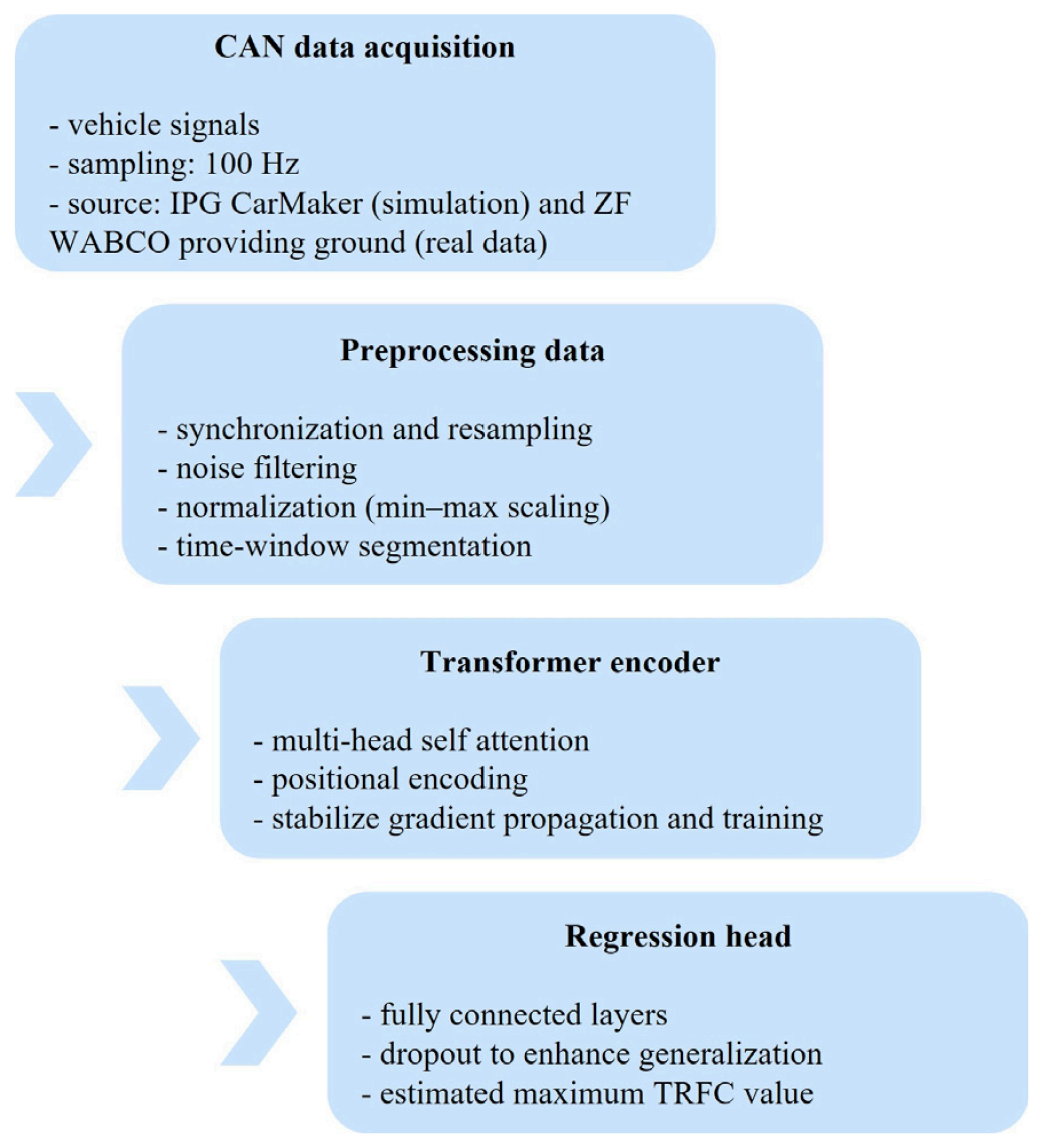
Transformer-based TRFC estimation from onboard CAN data, based on [65].

The use of Transformer Neural Networks (TNNs) has opened a new direction in the estimation of the maximum-friction coefficient, leveraging the self-attention mechanism to process CAN data sequences (Figure 13) more efficiently than recurrent architectures such as Long Short-Term Memory (LSTM) or Gated Recurrent Unit (GRU) [65]. Unlike these models, TNNs analyze the entire sequence simultaneously, reducing both training time and the number of parameters.

This advantage parallels improvements reported in image-dynamics-integrated estimation [103] and ANN-based road-surface classification [59], both consistent with the broader shift toward deep-learning architectures in TRFC-related estimation tasks.

The tests conducted in [65] on a Dacia Duster SUV, using both simulated data (IPG CarMaker) and experimental data (ZF WABCO proving ground), demonstrated that the TNN achieved median MSE values of 1.5·10^−4^ (longitudinal) and 1.7·10^−4^ (lateral) on real-world data, corresponding to an RMSE of approximately 0.012 ÷ 0.013, outperforming methods based on the Unscented Kalman Filter (UKF), GRU, and LSTM. Moreover, the TNN responds rapidly to abrupt changes in the maximum friction coefficient (e.g., wet–dry transitions), indicating more robust predictions when the dynamic conditions vary. However, its high sensitivity and lack of recurrence can lead to deviations in low-excitation regimes, requiring fusion with additional monitoring algorithms.

Nevertheless, the TNN achieved better accuracy than the baselines with fewer parameters and training epochs than the recurrent network baseline, although its real-time capability still has to be investigated [65].

### 5.6. Fusion of Computer Vision with Dynamic Models for Friction-Coefficient Estimation

**Figure 14:**
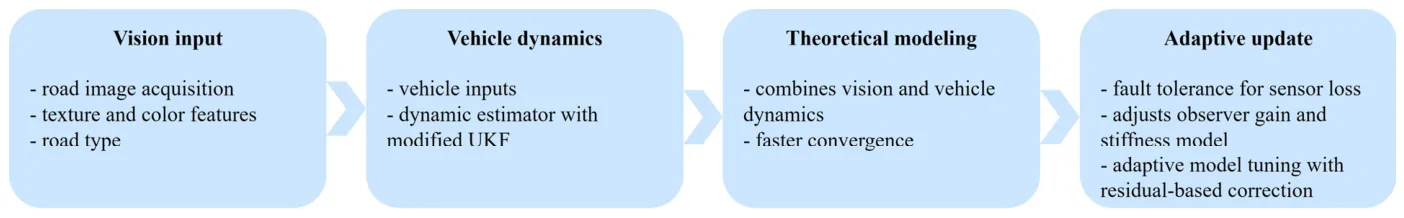
Compact framework of vision-dynamics fusion for TRFC estimation, based on [103,104].

The integration of computer vision with wheel dynamic models (Figure 14) has become an essential direction for the robust estimation of the TRFC. Purely dynamic models provide accurate results only under strong excitation, while vision-based approaches are sensitive to illumination conditions, making hybrid solutions advantageous [103,104].

A significant framework was proposed in [104], where camera images were processed through texture extraction (color moments and gray-level co-occurrence matrix), classified using Support Vector Machines (SVMs), and subsequently correlated with a modified Burckhardt observer. The visual result is employed to guide the gain of dynamic estimation, thereby accelerating convergence. In tests on an electric vehicle with independently driven motors, the fusion estimator converged in about 0.5 s, whereas the dynamics-only estimator did not converge for a long time under insufficient excitation. Moreover, under low-slip conditions the fusion estimator showed better accuracy and faster convergence than a dynamics-only estimator.

In a complementary direction, paper [103] proposed an adaptive fusion framework based on multi-temporal image fusion and a visual Transformer that recognizes the road surface at the wheel level. The visual result is integrated into an adaptive residual Unscented Kalman Filter (UKF), capable of detecting faults and adjusting its steady-state values in real time. Tests on real vehicles demonstrated an overall accuracy of 96.1% in surface classification and robust estimation of the friction coefficient even under extreme conditions (muddy post-snowfall roads, icy surfaces), while maintaining algorithmic stability in cases of model distortion or temporary loss of visual information.

As observed in these cases [103,104], vision–dynamic fusion provides a promising path toward adaptive estimation, which may enable faster and more robust predictions, particularly for electric and autonomous vehicles.

## 6. Integration of Interaction–Structure–Sensors

**Figure 15:**
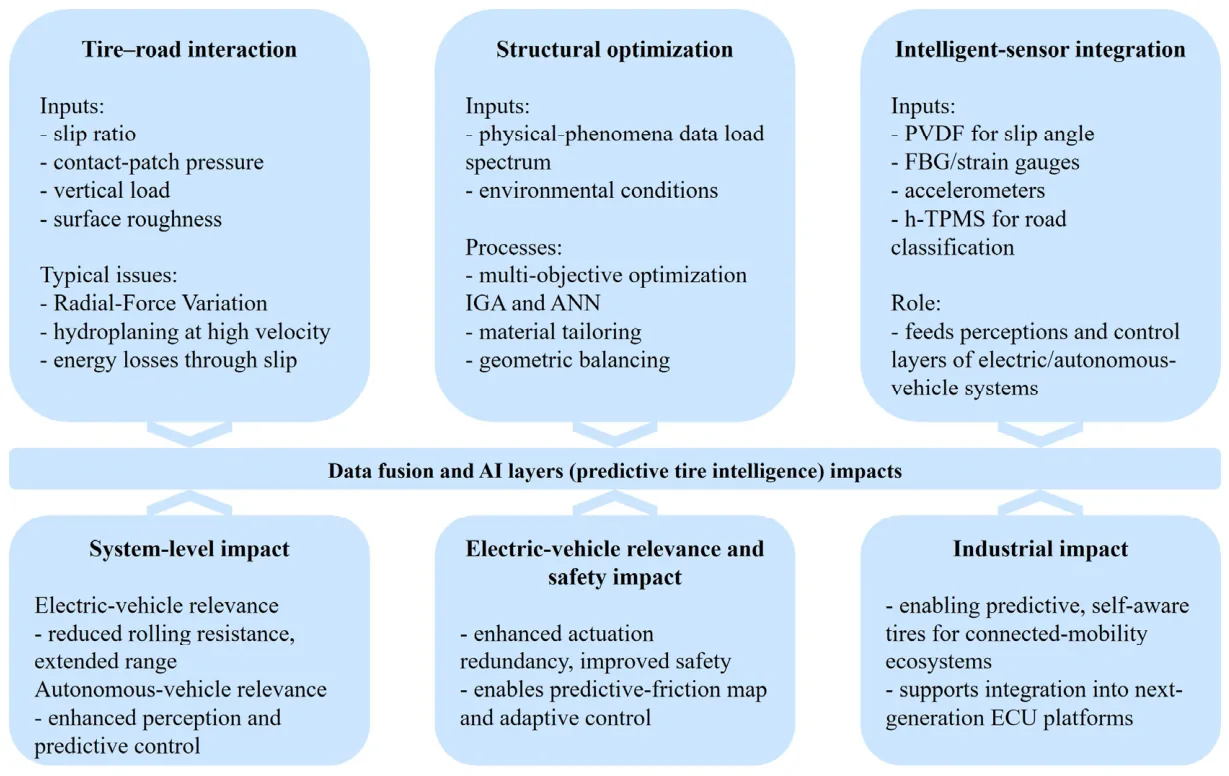
Integrated framework linking tire–road integration, structural optimization, and intelligent sensing for predictive tire development.

The need for an integrated framework for tires arises from the fact that physical phenomena, structural optimizations, and intelligent sensors cannot be treated in isolation if practical applicability to electric and autonomous vehicles is to be achieved (Figure 15).

Fragmented analyses in the current literature highlight progress in each direction, reducing energy losses through slip control [78], increasing adhesion via innovative composites [13], or estimating the friction coefficient using neural networks [65,103]. However, these contributions remain decoupled, which limits their industrial transferability.

In electric vehicles, where the total mass is higher [13] than that of conventional vehicles and range depends on the optimization of rolling resistance, as well as in autonomous vehicles, where safety relies on predictive perception, a holistic model is required, one that correlates tire–road interaction, structural design, and active sensor integration.

A clear example of the interdependence between physical phenomena, structural design, and sensor integration is the radial force variation (RFV), which generates additional excitations and reduces ride comfort [87]. Multi-objective optimizations assisted by genetic algorithms and ANN can mitigate this effect [88], but validation requires integrated sensors to measure local deformations [96]. Similarly, aquaplaning, determined by speed, pressure, and water film depth [29], can be delayed through design solutions, but predictive control appears to require real-time monitoring of forces [93].

A third example illustrates the same interdependence from the material side. In ref. [13], the HT compound showed the lowest wet-grip indicator (loss factor at 0 °C) and a higher rolling-resistance indicator (loss factor at 60 °C) than the TDAE reference, whereas STPB combined a wet-grip indicator close to the reference with a lower rolling-resistance indicator, illustrating that material-level structural choices constrain the operating envelope within which sensing and estimation must perform. This dependency is directly quantifiable at the sensor level. PVDF-based slip angle estimation maintains an average error below 5.5% under standard conditions, but degrades to a mean absolute percentage error of 8.89% on low-friction wet surfaces (μ = 0.4), compared to 5.42% on dry pavement. The maximum error exceeds 20% at slip angles beyond 4°, where the tire enters a nonlinear operating regime that distorts the sensor signal [97]. The resulting friction estimate then defines the activation threshold for ESC and ABS, with direct implications for autonomous vehicles, where reliable low-adhesion detection underpins perception redundancy in adverse weather. These three cases are summarized in Table 14, which maps the structural, sensing, estimation, and control-level interdependencies discussed above. Only the third case admits a quantitative estimation metric. For RFV and aquaplaning the corresponding cells record the mitigation strategy reported in the sources.

The role of sensors and artificial intelligence is therefore complementary to structural optimization. Technologies such as PVDF sensors for slip-angle detection [97], integrated accelerometers [95], or h-TPMS systems capable of surface recognition [100,101] transform the tire from a passive component into an active perception unit, providing critical variables for control systems. Furthermore, AI-assisted multi-sensor fusion [103,104] stabilizes and adapts predictions under variable conditions.

These directions converge toward the concept of the predictive tire, which integrates structural optimization, innovative composites [13] and active sensor integration to support both electric and autonomous vehicles. The value of this convergence lies not in the individual performance of each component but in the emergent capability that arises when structural parameters, sensing outputs, and estimation algorithms are co-designed. This integrated perspective appears to be necessary for the tire to fulfill its emerging role as an active node within the vehicle’s perception and control architecture.

## 7. Perspective on Predictive Tires for Electric and Autonomous Vehicles

**Figure 16:**
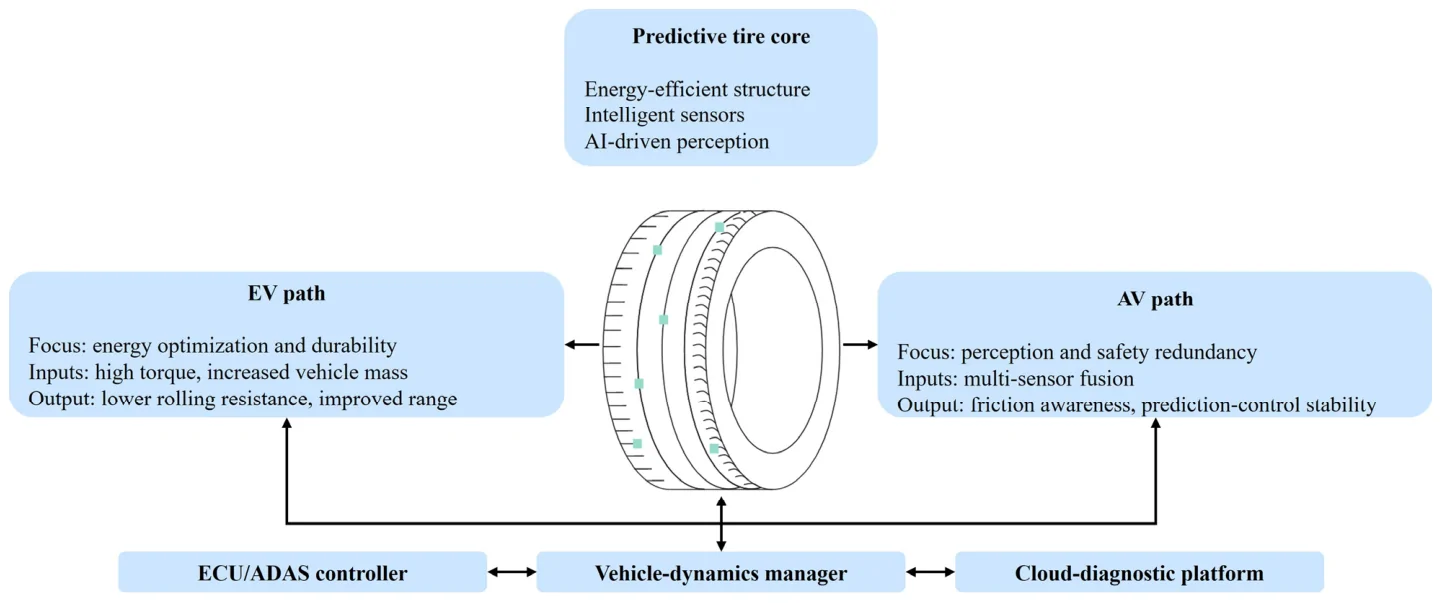
Conceptual roadmap of predictive tire integration within electric and autonomous vehicle architectures. Green dots indicate the locations of sensors embedded within the tire structure.

The evolution of the tire toward a predictive role opens a development framework with distinct implications for electric and autonomous vehicles (Figure 16). In the case of electric vehicles, the focus shifts beyond safety toward balancing energy efficiency and durability. The increased mass and instantaneous torque amplify the mechanical stresses on the tire, making the optimization of materials and internal architecture a decisive factor for range performance [20,50,57,78].

Beyond energy efficiency and perception redundancy, predictive tire functions translate directly into two vehicle-level control metrics common to both electric and autonomous platforms: braking performance and vehicle stability. Accelerometer-based intelligent tires have been shown to detect vibrations indicating sliding in the contact patch, already visible at about 3.7% brake slip in the maneuver analyzed in [95], offering a potential additional input for ABS control beyond wheel-speed-derived estimates. At the friction-estimation level, a sensor-free particle-filter approach validated in braking, acceleration, and sinusoidal steering tests (including ABS-activated braking on a split-μ road) has achieved friction coefficient RMSE values of at most 0.037 [73]. The corresponding lateral force and sideslip angle RMSE values were up to 66 N and 0.08°, respectively, a level of precision directly relevant to electronic stability control. Control-allocation strategies explicitly formulated around the joint objective of minimizing tire-slip energy and maintaining vehicle stability [78] further illustrate that these two vehicle-level outcomes are not independent design targets but are shaped jointly by the same tire–road interaction variables discussed throughout this review.

For autonomous vehicles, the tire assumes a strategic function within the perception architecture. Unlike optical sensors, which can be limited by weather or visibility conditions, the tire provides direct data regarding the state of the road surface and the actual limits of adhesion. Thus, it can act as a complementary sensor to LiDAR, radar, or cameras by supplying otherwise inaccessible variables such as the friction coefficient, the evolution of forces generated in the contact patch, or even early signs of aquaplaning. Integrating these strategic control data enhances vehicle safety in unpredictable scenarios [18,19,60].

Looking forward, the most promising direction is not the accumulation of isolated sensors but the convergence of structural design, distributed sensor integration, and predictive algorithms. The tire is no longer merely a component responsible for transmitting and absorbing the forces generated at the road interface but becomes an informational node integrated into the vehicle architecture [22,93,105]. This points to a stage in which the tire may become an essential part of sustainable and automated mobility infrastructure, contributing to both extended range for electric vehicles and robust safety for autonomous vehicles.

## 8. Current Challenges

Despite the remarkable progress in intelligent and predictive tire research, several major limitations still hinder large-scale adoption. These challenges arise from both technological constraints and the lack of standardized frameworks, affecting the reliability and transferability of current solutions. As a result, bridging the gap between experimental developments and industrial implementation remains a critical objective.

### 8.1. Lack of Standardized Testing and Validation Protocols

A major obstacle to the industrial transferability of intelligent tire technologies is the absence of unified testing frameworks that span both laboratory and real-world driving conditions. As explicitly identified by [22], the development and validation of intelligent tire systems currently face critical challenges precisely because no integrated bench-to-road testing protocols exist, forcing researchers to design ad hoc validation procedures that are incompatible across laboratories and vehicle platforms. This fragmentation makes it difficult to reliably compare results obtained through different sensor configurations, road surface conditions, or vehicle dynamics setups, undermining confidence in the scalability of reported performance metrics.

At the algorithmic level, paper [18] highlights that current intelligent tire sensing systems, while effective in controlled scenarios, have not been validated under the continuously variable and extreme working conditions inherent to real tire operation, and that the lack of standardized sensor design guidelines limits reproducibility across independent research groups. Similarly, the authors of [19] identify the absence of comprehensive, representative datasets as the primary constraint on the generalization capability of data-driven TRFC estimation methods, noting that most existing algorithms are trained on narrow, controlled datasets that fail to capture the full variability of real-world driving conditions. Consequently, model performance has been reported to degrade when transferred to unseen road surfaces, vehicle types, or climatic conditions, a limitation that may compromise the reliability of predictive tire architectures in safety-critical applications.

### 8.2. Production Costs and Integration Complexity

The transition from laboratory prototypes to series-production intelligent tire systems is further constrained by the high cost and engineering complexity associated with the direct integration of sensing elements into the tire structure. Unlike conventional automotive sensors mounted externally, embedded tire sensors must withstand continuous mechanical deformation, thermal cycling, and chemical exposure, requirements that current manufacturing processes are not yet optimized to meet at scale. The authors of [23] explicitly note that sensor-based approaches for direct tire–road interaction measurements are difficult to implement on production vehicles due to both the cost of the sensing hardware and the engineering challenges of maintaining signal integrity within the mechanically and thermally aggressive environment of a rotating tire. This observation, made in the context of friction coefficient estimation for electric vehicles, reflects a broader challenge. The economic viability of embedded sensing solutions has not yet been demonstrated at the scale required for mass-market deployment, particularly given the cost sensitivity of the automotive supply chain [23,61].

Beyond unit cost, the integration complexity of multi-sensor architectures, combining accelerometers, strain gauges, PVDF films, and wireless transmission modules within a single tire structure, introduces additional challenges in terms of assembly processes, electromagnetic compatibility, power management, and calibration procedures. These factors represent significant barriers to OEM adoption, particularly in the absence of standardized integration protocols and homologation frameworks.

### 8.3. Long-Term Durability and Reliability Under Service Conditions

Perhaps the least addressed challenge in the current literature concerns the long-term durability of embedded sensing elements across the full service life of a tire. Paper [106] demonstrates that flexible sensors based on laser-induced graphene can successfully monitor tire deformation and road interaction in real time, but explicitly acknowledges that maintaining sensor performance under the cyclic mechanical stress, thermal variation, and chemical exposure characteristics of tire operation over tens of thousands of kilometers remains an open challenge. This limitation is not unique to graphene-based sensors; it applies equally to PVDF films [97], FBG strain sensors [93], and integrated accelerometers [95], none of which have been subjected to systematic long-term durability testing across a complete tire service life cycle in the published literature [107].

The durability problem is compounded for electric vehicles, where higher static loads, elevated instantaneous torques, and regenerative braking cycles impose more severe and asymmetric mechanical demands on the tire structure than those encountered in conventional powertrain configurations. Under these conditions, the adhesion between sensing elements and the rubber matrix, the integrity of embedded wiring, and the calibration stability of sensor outputs are all subject to accelerated degradation mechanisms that have not yet been quantified experimentally [61].

Bridging the gap between experimental demonstrations and industrial implementation requires progress along three parallel tracks: standardized multi-condition validation protocols [103], the construction of open representative datasets for AI-based estimator training and benchmarking [23], and the systematic characterization of embedded sensor durability across full tire service life cycles [107]. Until these foundations are established, the predictive tire concept is likely to remain confined to laboratory demonstrations, regardless of the algorithmic and structural advances documented in the preceding sections.

### 8.4. A Staged Validation Pathway and Development Roadmap Toward Practical Deployment

Addressing the challenges identified in Section 8.1, Section 8.2 and Section 8.3 requires progress to be organized along an explicit, staged validation pathway, moving from component-level characterization to full vehicle-level integration. In the short term, priority should be given to sensor and material-level characterization under controlled bench conditions, complemented by the construction of open, representative datasets and shared benchmarks that would allow different sensing and estimation approaches to be compared on a common basis, directly addressing the fragmentation identified in Section 8.1. In the medium term, validation should extend to multi-condition tire-rig testing under systematically varied loading and environmental conditions, including cross-domain bench configurations capable of jointly evaluating the structural, sensing, and estimation interdependencies summarized in the matrix of Table 14. This should be followed by multi-platform real-vehicle trials spanning different vehicle classes and powertrain architectures, alongside dedicated life-cycle and durability testing protocols capable of reproducing the cyclic mechanical, thermal, and chemical stresses discussed in Section 8.3. At this stage, compliance with functional safety standards becomes relevant, because predictive tire outputs feed directly into ABS, ESC, and ADAS control loops, as discussed in Section 7. Such outputs would typically require classification under an Automotive Safety Integrity Level (ASIL) framework, such as ISO 26262:2018 [70], before deployment in series-production safety-critical systems. Emerging automotive cybersecurity requirements become similarly relevant as tire-derived data increasingly feeds connected vehicle architectures. Existing regulatory instruments already governing tire-related sensing, such as UNECE Regulations No. 64 and No. 141 for tire-pressure monitoring [98,99], illustrate that formal validation and certification pathways for embedded tire sensing are not without precedent, though their scope has not yet been extended to the broader predictive-tire functions reviewed in this work. In the long term, the field is expected to move toward self-powered sensing architectures that reduce dependency on wired power delivery within the rotating tire structure, and toward digital-twin representations of individual tires capable of predicting tire health and durability under varying operating and environmental conditions [107]. The adoption of sustainable materials selected through explicit life-cycle assessment (LCA) would further extend the composite-material discussion of Section 3.4 beyond performance metrics alone.

This staged pathway also differs in emphasis between electric and autonomous-vehicle platforms. For electric vehicles, validation priorities center on rolling resistance, driving range, and tire wear under the elevated static and dynamic loads discussed in Section 8.3, given their direct link to energy consumption and total cost of ownership. For autonomous vehicles, validation priorities instead center on perception redundancy, fault tolerance, and the stability of predictive control outputs under sensor degradation or partial failure, reflecting the tire’s emerging role as a safety-critical perception node discussed in Section 7. Such a staged, dual-track pathway, spanning sensor and material characterization, tire-rig and vehicle validation, durability assessment, multi-condition testing, and integration with ABS, ESC, and ADAS, is likely to be a necessary condition for transitioning predictive tire technologies from the laboratory-scale demonstrations documented throughout this review toward certified, mass-produced automotive systems.

### 8.5. Electromagnetic Compatibility and Calibration

Two further practical constraints deserve explicit discussion. Calibration recurs throughout the reviewed literature, from the finite-element-to-physical correlation [88,94,96] to sensor-specific calibration against geometric and thermal effects [95,97] and statistically calibrated uncertainty in data-driven estimators [33], indicating that calibration is not a peripheral concern but a cross-cutting requirement for the methods surveyed. Electromagnetic compatibility (EMC), by contrast, is entirely absent from the reviewed intelligent-tire literature. This is a significant gap: EMC is a well-established concern for automotive electronic systems more broadly [71], where interference has been shown to degrade sensor accuracy and compromise safety-critical control functions such as ABS and ESC. Given that predictive tire architectures increasingly combine wireless data transmission with proximity to safety-critical control loops, the absence of EMC characterization for embedded and wireless tire sensing systems represents an unaddressed risk that warrants dedicated investigation before large-scale deployment.

## 9. Discussions

The four domains examined in this study, wheel–road interaction, tire structural design, smart sensing architectures, and multimodal friction coefficient prediction, appear to be interdependent. A discussion should go beyond the individual analyses and highlight how these domains may converge to define the emerging paradigm of predictive tires. The cross-domain dependencies identified throughout this review suggest that isolated advances in any single direction have limited transferability unless they are explicitly connected to the constraints and requirements of the other three domains.

Across the primary studies underpinning the synthesis presented in Table 2, Table 3, Table 4, Table 5, Table 6, Table 7, Table 8, Table 9, Table 10, Table 11, Table 12, Table 13, Table 15, Table 16 and Table 17, validation approaches differ substantially in physical fidelity. Of the 23 distinct studies classified in these Table 2, Table 3, Table 4, Table 5, Table 6, Table 7, Table 8, Table 9, Table 10, Table 11, Table 12, Table 13, Table 15, Table 16 and Table 17, four report results derived exclusively from numerical simulation, without any physical validation component, and nine rely solely on physical testing at the bench, tire-rig, or vehicle level. Another nine combine a simulation stage with subsequent physical validation, most commonly to confirm a finite element or control model before extending it to experimental conditions, and one [73] was validated through real-time Hardware-in-the-Loop simulation. This heterogeneity has a direct implication for the interpretation of the performance metrics compiled throughout this review: accuracy and error figures reported under purely simulated conditions are not directly comparable to figures obtained under tire-rig or on-road conditions, where sensor noise, thermal drift, and unmodeled road irregularities typically degrade performance relative to simulation. This heterogeneity is already visible in the comparative analysis of Section 9.4, where model-based estimators are noted to remain superior under high-excitation conditions, while AI-based approaches tend to perform better in noisy or low-slip scenarios, a pattern consistent with differing validation regimes rather than differing algorithmic merit alone. Readers should therefore treat cross-study comparisons of reported accuracy with corresponding caution, and the absence of standardized testing and validation protocols, discussed further in Section 8.1, remains a central barrier to establishing performance benchmarks that are transferable across the domains covered in this review. Notably, none of the 23 classified studies report validation under genuine real-road conditions with uncontrolled traffic. Where vehicle-level testing is reported, it was conducted on closed test tracks or dedicated proving grounds, which suggests that real-road validation remains an open gap in the reviewed literature.

### 9.1. Wheel–Road Interaction

Integrating the insights from [29,34,78] provides a concrete overview of wheel–road interaction. Energy losses through slip can be reduced through predictive methods such as MPCA, with potential benefits for range and sustainability, as indicated by simulation results [78]. The contact patch is sensitive to load and speed, especially on wet roads, where the contact length decreases markedly as the speed approaches the aquaplaning limit [34]. Aquaplaning remains a critical limitation, where high pressure can delay the phenomenon, but wear and severe road conditions accelerate it [29].

Seen together, these results indicate that physical interaction alone may not ensure robust performance in electric and autonomous vehicles unless it is paired with real-time sensing and adaptive control. This supports the case for integrated sensing-control strategies within the tire itself.

### 9.2. Structural Optimization and Advanced Materials

Literature analysis [13,87,88,90] indicates the convergence of several directions, such as radial stiffness control for NVH, multi-objective optimization assisted by artificial intelligence, multi-physics modeling, and the introduction of innovative composite materials. The results are summarized in Table 15, which highlights the main structural innovations, their impact on vehicle dynamics, and their associated limitations.

**Table 15:** Summary of structural innovations in tires, data from [13,87,88,90].

Structural Innovation	Innovative Character	Impact on Vehicle Dynamics	Limitations/ Challenges	Validation Level
Radial-force variation	Controlling stiffness and unevenness through advanced manufacturing processes	NVH reduction, increased comfort, improved stability	Advanced control of the manufacturing process	Numerical simulation
Multi-objective optimization	Artificial intelligence models for structural parameters	Increased durability, improved stability, reduction of critical loads	High computational complexity	Numerical simulation
Integration of deformation, contact analysis, and aerodynamics (ST-SI-TC-IGA)	Unified space–time framework with isogeometric analysis	High-fidelity representation of tire deformation and road contact	Purely computational; no quantified accuracy metrics reported	Numerical simulation
Open-structure silica network (Silane-Terminated Telechelic Polybutadiene)	Innovative molecular architecture for composites	Grip-efficiency balance, longer EV range, less wear	Sensitivity to temperature and humidity variations	Bench testing

Therefore, controlling radial variation can improve handling at high speeds, but requires advanced manufacturing technologies. At the same time, multi-objective optimization based on evolutionary algorithms and neural networks has brought notable predicted gains in durability, but is limited by computational complexity. The ST-SI-TC-IGA framework offers a high-fidelity computational representation of tire deformation and road contact, but so far without quantified accuracy metrics, while Silane-Terminated Telechelic Polybutadiene promises a favorable balance between grip and energy efficiency, with increased sensitivity to temperature and humidity variations.

Thus, although each direction has individual potential, the greatest value appears to emerge from their integration into a unified framework, where the optimized structure is validated through multi-physical simulations and supported by innovative materials, paving the way for the integration of smart sensors and friction coefficient prediction, which will be discussed in the following sections.

### 9.3. Smart-Tire Architecture

Critical analysis of different sensor technologies indicates the modern tire’s evolution toward the role of a multifunctional platform, capable of providing both mechanical information (forces, moments, stiffness) and real-time status parameters (coefficient of friction, road surface condition, tire wear). Progress is evident from unitary sensors (deformation, accelerometers) to hybrid solutions based on AI and neural networks, which can support a more robust estimation of wheel–road interaction.

Table 16 summarizes these results, highlighting how each method contributes specifically to improving vehicle safety and dynamic behavior, but also the current limitations in terms of cost, long-term reliability, and lack of industry standardization. These systems outline the transition to the smart tire, integrated into the control architecture of electric and autonomous vehicles.

The unifying observation is that no individual sensing technology appears sufficient for predictive control. Instead, their combined use, together with machine-learning fusion, is likely to form the basis of predictive tires. This aligns smart sensing with structural and friction-prediction requirements.

**Table 16:** Overview of sensor technologies integrated in tires.

Sensor Type/Method	Estimated Parameter	Impact on Vehicle Dynamics	Challenges/Limitations	Validation Level
Unitary strain sensors (FBG) [93]	Tire deformation, F_x_/F_y_ indirect	Direct correlation with contact patch for estimating longitudinal/lateral forces	High sensitivity to noise, difficult to install in tires	Vehicle test
ANN + fuzzy fusion [8]	Estimating normal force using hybrid methods	Improved ABS/ESC control elements, stability maintained on variable surfaces	Requires extensive training data	Numerical simulation + vehicle test
Normal force-estimation algorithm [94]	Real-time normal force	Estimation of normal forces for suspension control and torque vectoring	Accuracy decreases during extreme maneuvers	Numerical simulation + tire-rig experiment
Integrated accelerometers [95]	Transition between adhesion and sliding	Stability loss detection	Accelerometers may deviate under extreme vibrations and temperatures	Vehicle test
Integrated strain gauges [96]	Normal force, slip	Estimating grip, adjusting braking	Low costs, but limited durability	Numerical simulation + vehicle test
PVDF piezoelectric sensors [97]	Direct estimation of the slip angle	Lateral control, cornering stability	Temperature sensitivity	Numerical simulation + tire-rig experiment
Triaxial force sensor mounted on the belt [32]	Simultaneous measurement of F_x_, F_y_, F_z_ on a single tread block	Detailed picture of tread-block/road interaction	Complex structural integration (belt, drilled through), calibration against belt deformation required	Tire-rig experiment
h-TPMS + CNN [59,100,101]	Wear detection through neural networks, pressure, and road-surface type	Adaptive monitoring, surface classification, AV/EV safety	Requires large databases and periodic recalibration	Vehicle test + tire-rig experiment

### 9.4. Multimodal Friction Prediction

The comparative analysis of friction-coefficient prediction methods, summarized in Table 17, highlights the transition from dynamic models validated by real-time Hardware-in-the-Loop simulation for electric vehicles with in-wheel motors [73] to data-driven frameworks with uncertainty evaluation [33], which provide confidence intervals but have so far been validated experimentally only on wet and dry asphalt.

The combined approaches involving camber angle, slip angle, and wheel load [66] contribute to the calibration of control systems but remain confined to controlled scenarios. Hybrid methods (Kalman and fuzzy) [62] and Transformer networks [65] are reported to provide accurate and stable predictions, albeit with computational demands described in relative terms [62,65], while the fusion of visual information with wheel dynamics [104] extends practical applicability but introduces illumination-related challenges.

A deeper critical comparison across these methods indicates substantial heterogeneity in the rigor of uncertainty quantification and in computational cost. The SVDKL framework [33] is the only method among those compared here to provide a statistically calibrated uncertainty measure, via a sparse variational Gaussian process that yields a genuine confidence interval around each point estimate. A weaker form of confidence quantification appears in [103], where the image-based sub-estimator reports an explicit confidence signal (exceeding 90% under favorable visibility, dropping to 50% under occlusion) that informs the adaptive fusion behavior, though this is a heuristic classifier-derived signal rather than a formally calibrated statistical interval. The particle-filter-based estimator [73] occupies an intermediate position: although particle filters inherently carry a distributional representation of the state estimate from which uncertainty could in principle be derived, this capability is not reported or exploited in the cited evaluation, which presents only point estimates and RMSE values. By contrast, the Transformer-based estimator [65] is explicitly described by its authors as a black-box model providing no uncertainty measure, and the PCKF-SOAFNN scheme [62] likewise reports only point estimates. Regarding computational burden, concrete figures remain scarce but instructive where reported. The PVDF-based slip angle estimator [97] required 92.12 s to train and 1.1 ms per inference for its best-performing Gaussian process regression model, against 135.21 s and 2.1 ms for a multilayer perceptron, and 88.24 s and 1.0 ms for a random forest on the same dataset. For the image-based fusion estimator [104], image classification alone accounts for most of the computational cost, at 0.1 ÷ 0.2 s per frame on a standard industrial PC. The particle-filter-based estimator [73] achieved a response time of 535 ms on a processor equivalent to mainstream production vehicle control units, against 567 ms for an EKF baseline evaluated on the same platform, indicating both compatibility with hardware constraints and a measurable, if modest, efficiency advantage over the classical alternative. By comparison, the computational costs attributable to the hybrid Kalman-fuzzy [62] and Transformer-based [65] estimators are reported only in relative terms (fewer parameters and a smaller network size than alternative architectures), rather than as absolute training or inference times. The qualitative characterization of these methods as computationally demanding should therefore be read as a comparative, not an independently quantified, claim.

Regarding performance under unseen or extreme conditions, the evidence is mixed but instructive. The PVDF-based estimator [97] shows its MAPE rise from 5.47% under standard cross-validation to 7.16% under extreme maneuvers, with error exceeding 20% at slip angles beyond 4° on wet surfaces. Generalization to unseen tire conditions was also directly tested for this method: with tread wear varied between 0 and 4 mm, the MAPE shifted only modestly (7.16% at zero wear versus 8.31% and 7.52% at 2 mm and 4 mm, respectively). This indicates that this particular estimator is comparatively robust to tire-condition variation, even as its performance under demanding road conditions degrades more substantially. The Transformer estimator [65] is reported to produce larger outliers under low vehicle dynamic excitation, and the PCKF-SOAFNN scheme [62] is explicitly acknowledged by its authors as unable to handle complicated or bumpy road profiles. A closely related limitation affects purely dynamics-based estimators more broadly: both the particle-filter estimator [73] and the fusion estimator of [104] identify near-constant-speed, low-excitation driving as a specific failure mode for dynamics-only estimation. In ref. [104], a dynamics-only estimator failed to distinguish road surfaces under these conditions, while the corresponding vision-augmented fusion kept the error within ±0.05. Against this pattern, the image-based estimator of [103] was tested exclusively on data withheld from training and maintained over 95% accuracy across six distinct real-world surface conditions, including wet asphalt, post-snow mud, and icy roads. It was also stress-tested under deliberately induced tire-pressure loss to simulate model mismatch, indicating broader generalization evidence than is reported for the other methods discussed above. Taken together, these findings indicate that none of the AI-based methods reviewed here simultaneously provides calibrated uncertainty, low computational cost, and robust generalization to unseen road and tire conditions, and that low-excitation, near-constant-speed driving remains a specific, recurring weakness for dynamics-only architectures that visual or multimodal fusion approaches appear best positioned to address. It should also be noted that, beyond the PVDF-based estimator [97], generalization evidence explicitly addressing unseen tire conditions, such as wear, pressure, or dimensional variation, remains scarce across the reviewed AI-based methods.

Overall, the diversity of the analyzed methodologies suggests that friction coefficient prediction is evolving toward a multimodal architecture. In such an architecture, the combination of dynamic models, AI algorithms, and sensor fusion can transform the tire from a passive component into an active node of perception and control, with a potentially central role in the stability, maneuverability, and safety of electric, autonomous, and ADAS-equipped vehicles.

The comparison across TRFC estimation methods highlights a complementarity: model-based estimators tend to remain superior under high-excitation conditions, while AI-based approaches show a tendency to perform better in noisy or low-slip scenarios. This suggests that future predictive tires may benefit from a hierarchical estimator, combining physical modeling, AI inference, and visual cues with dynamically weighted confidence levels.

The synthesis suggests that predictive tires are unlikely to emerge from isolated innovations. Their development appears to require a systemic convergence of structural optimization, multimodal sensing, and uncertainty-aware friction prediction. The literature consistently reports progress within each domain, yet the lack of unified frameworks appears to be the main barrier to real-world deployment. This integrated perspective provides a conceptual anchor linking Section 7 and Section 8, and outlines a possible roadmap toward practical implementation.

**Table 17:** Synthesis of the methodologies regarding the prediction of the friction coefficient.

Methodology	Innovation Character	Impact on Vehicle Dynamics	Limitations/ Challenges	Validation Level
Friction coefficient estimation through particle-filter-based dynamic models on electric vehicles with in-wheel motors [73]	Direct integration into electric vehicles; Hardware-in-the-Loop validation under scenarios involving complex maneuvers	Improve vehicle control systems such as ABS and ESC, by adapting to varying adhesion conditions	Validated only by real-time Hardware-in-the-Loop simulation; no on-road tests	Numerical simulation (real-time Hardware-in-the-Loop)
Data-driven framework with optimal data-set selection and uncertainty evaluation [33]	Introduction of robust uncertainty assessment in friction-coefficient estimation	Provides confidence intervals for the estimate and lower errors than the compared methods at low excitation	Experimentally validated on wet and dry asphalt only; larger errors in nonstationary maneuvers on high-friction asphalt	Numerical simulation + vehicle test
Analysis of combined interaction: slip angle and camber angle, with effect on contact patch [66]	Study explicitly dedicated to combined conditions of slip and camber angles	Enables calibration of ESC and lateral models under critical conditions	Based on tire-rig and model results; no vehicle-level or real-time implementation reported	Numerical simulation + tire-rig experiment
Enhanced models applied to vehicles with independent steering and propulsion [62]	Distributed estimation of the friction coefficient	More accurate friction estimation than the CKF- and RLS-based baselines in the tested conditions	Cannot deal with complicated or bumpy road profiles (acknowledged by the authors)	Numerical simulation + vehicle test
Application of Transformer networks on sensor-based data [65]	Study applying Transformer networks to estimate the maximum friction coefficient value	Faster and more accurate response to jumps in the maximum-friction coefficient than the UKF baselines	Larger outliers under low dynamic excitation; black-box model; real-time capability not yet evaluated	Numerical simulation + vehicle test
Fusion of camera-based road classification and tire dynamics [104]	Does not require direct tire–road interaction sensors	Faster convergence of the peak friction coefficient estimate under low excitation	Sensitive to lighting conditions and image quality	Vehicle test
Wheel-level multi-temporal image fusion with an adaptive UKF [103]	Adaptive method for variable road conditions	Supports stable and reliable prediction for ESC/ADAS control	Performance depends on image quality (confidence drops under occlusion)	Numerical simulation + vehicle test

### 9.5. Method-Level Traceability of Dataset, Scenario, and Readiness Level

To situate the reviewed methods along a common maturity scale, this synthesis adopts the nine-level Technology Readiness Level (TRL) framework as defined by the European Commission [72], mapped onto the five-tier validation categories used consistently throughout the tables of this review. Numerical simulation corresponds to TRL 3 (experimental proof of concept), bench testing to TRL 4 (technology validated in lab), tire-rig experiments to TRL 5 (validated in an industrially relevant environment), and vehicle tests to TRL 6 (demonstrated in a relevant environment). Real-road validation, involving uncontrolled traffic and public road conditions, would correspond to TRL 7 (system prototype demonstration in operational environment). Hardware-in-the-Loop implementations, in which only the control hardware, not the tire or road interface, is physical, are conservatively assigned TRL 4, reflecting partial rather than full satisfaction of the “relevant environment” criterion. Consistent with the absence of real-road validation identified earlier in this review, none of the methods synthesized in Table 18 reach TRL 7 or above.

## 10. Conclusions

The critical analysis conducted in this study indicates that the tire is increasingly regarded as a multifunctional component with an active role in the vehicle’s control architecture, rather than as a simple passive element for force transmission. The analysis of wheel–road interaction highlighted key phenomena such as energy losses due to slip, aquaplaning, and radial-stiffness variation, all of which influence vehicle stability, handling, and energy efficiency.

In parallel, the reviewed structural optimizations indicate that multi-objective design algorithms and innovative composite materials can contribute to reducing rolling resistance and improving NVH performance, while maintaining safety-relevant performance in the cases studied.

The integration of intelligent sensors points to a transition toward a new tire model, a sensor-equipped tire, capable of providing real-time critical parameters such as longitudinal and lateral forces, normal load, slip angle, and friction coefficient. The prediction of the latter, through multi-sensor data fusion and advanced artificial intelligence algorithms, can open the way to more robust predictive control, which is important for electric and autonomous vehicles.

The physical phenomena described at the wheel–road interface appear to be best addressed through a combination of structural design and active sensor integration, supported by AI models capable of interpreting complex and variable data.

The main contribution of this work lies in illustrating, through the worked cases of Section 6, that wheel–road interaction, structural optimization, intelligent sensing, and TRFC estimation are interconnected layers of a predictive tire architecture rather than independent research directions. Structural parameters can constrain sensor integration, material properties can influence estimation accuracy, and sensing outputs can bound the performance of vehicle control systems.

The synthesis also identifies three main research gaps that should be addressed to enable industrial deployment. First, none of the studies reviewed here has simultaneously validated structural, sensing, and estimation performance on the same physical tire under identical operating conditions, so cross-domain experimental co-validation appears to be absent from the reviewed literature. Second, the AI-based TRFC estimation methods reviewed here have not been benchmarked against standardized real-world datasets spanning multiple vehicle platforms, sensor configurations, and climatic conditions. Third, the long-term durability of embedded sensing solutions, such as FBG, PVDF, and accelerometer-based systems, under the full thermal and mechanical cycling characteristics of tire service life has not been systematically quantified in the reviewed studies.

Building on the identified research gaps, the following directions are proposed as a concrete future research agenda. First, cross-domain experimental co-validation is needed, wherein structural, sensing, and TRFC estimation performance are simultaneously characterized by the same physical tire specimen under identical operating conditions, bridging the currently disconnected laboratory protocols. Second, the construction of open, standardized datasets spanning multiple vehicle platforms, sensor architectures, road surface types, and climatic conditions is important to enable reproducible benchmarking of AI-based TRFC estimators. Third, systematic long-term durability studies of embedded sensing elements, including FBG fibers, PVDF films, and MEMS accelerometers, should be conducted across the full thermal and mechanical cycling profile of a tire service life. Fourth, the integrated predictive tire architecture outlined in this work should be validated on in-wheel motor EV platforms under mixed driving conditions (urban, highway, and off-road), where the combined effect of higher static loads, regenerative braking, and instant torque delivery is likely to represent the most demanding operational envelope.

In conclusion, the future of predictive tires is likely to depend not only on the excellence of fundamental research but also on overcoming the barriers of cost, durability, and standardization. Strengthening an ecosystem of collaboration between manufacturers, researchers, and technology providers is an important prerequisite for transitioning from experimental prototypes to large-scale industrial implementation.

## Figures and Tables

**Table 1:** Comparative thematic coverage.

Reference	Wheel–Road Interaction	Structural Optimization	Intelligent Sensing	TRFC Estimation	EV/AV Focus	Cross-Domain Interdependency Analysis
[18]	✗	✗	✓	✓	Partial	✗
[19]	✗	✗	✗	✓	✗	✗
[20]	✗	✗	✓	✓	✗	✗
[21]	✗	✗	Partial	✗	✓	✗
[22]	✗	✗	✓	✗	✗	✗
[23]	✗	✗	✗	✓	Partial	✗
Present work	✓	✓	✓	✓	✓	✓

✓ = the domain is analyzed in depth, with dedicated methodological or quantitative content; ✗ = the domain is not addressed; Partial = the domain is mentioned in passing or covered only for a subset of vehicle types (e.g., EV or AV, but not both). The rightmost column indicates whether the source explicitly analyzes how the four domains constrain one another (e.g., how structural design bounds sensor placement, or how sensor output bounds estimation accuracy), rather than treating them as independent topics.

**Table 2:** Comparative analysis of methods studied for reducing energy losses, data from [78].

Method	Load Distribution	Longitudinal Energy Losses	Lateral Energy Losses	Directional Stability	Impact on EV Autonomy	Validation Level
TWUO	Increased demand on front axle	High	High	Low, risk of lateral saturation	Negative, with reduced autonomy	Numerical simulation
MPCA	Balanced redistribution	**↓** up to 50% vs. TWUO	**↓** ~35% vs. TWUO	Elevated, improved stability	Positive, with improved autonomy	

**↓** indicates a decrease and ↑ an increase relative to the reference condition stated in each row (e.g., TWUO).

**Table 3:** Synthesis of the effects of parameters (inflation pressure, vertical load, water depth, tire type) on the footprint and stability of the vehicle, data from [29].

Parameter	Effect on the Contact Patch	Dynamic Consequences	Observations	Validation Level
Inflation pressure ↑	Small contact patch, efficient water drainage	Critical threshold **↑** (better resistance to aquaplaning)	May reduce lateral grip on dry roads	Vehicle test + tire-rig experiment
Vertical load ↑	Contact patch **↑**, local pressure **↑**	Stability on dry road **↑** at low velocity, but the hydrodynamic film forms more quickly	Amplified effect at EV due to higher total mass	
Water-depth layer ↑	Drainage efficiency **↓**	Critical VDA threshold **↓**	Causes unstable transition, sudden increase in slippage	
Tire type (mud and snow)	Stiffness **↓**, deformation **↑**	Rapid increase in longitudinal slip ratio, maneuverability **↓**	Critical threshold **↓,** early aquaplaning	

**Table 4:** Effect of structural optimization on the peak strain energy density at the belt edge and the peak shear strain of the carcass of a truck and bus radial tire, data from [88].

Structural Model	Peak-Strain Energy Density at the Belt Edge [MPa]	Peak Shear Strain of the Carcass [-]	Validation Level
Initial design	0.363	0.064	Numerical simulation
Optimized	0.287	0.039	
Optimized, direct finite-element analysis	0.290	0.042	

**Table 5:** Comparative synthesis of composite materials used in the analysis and their impact on vehicle dynamics, data from [13].

Material	Mechanical Resistance [MPa]	Wear Resistance (Loss) [mg]	Cold Grip (0 °C, Loss Factor)	Rolling Resistance (60 °C, Loss Factor)	Impact on Vehicle Dynamics	Validation Level
TDAE	~18.1	~250	Satisfactory	High, significant energy losses	Acceptable stability, but high energy consumption	Bench testing
R131	~19.1	~231	Similar to TDAE	Similar to TDAE	Slight improvements in wear, with no major impact on safety	
HT	~19.4	~239	Lowest of the four	High, significant energy losses	Reduced wet-grip indicator, low efficiency	
STPB	~19.1	~225	Acceptable (comparable with TDAE)	Reduced, ~14 ÷ 16% lower loss factor vs. TDAE reference	Stability, grip, low energy consumption, and durability	

**Table 6:** Performance of models for estimating normal force and vehicle mass, data from [8].

Method	Principal Characteristics	Performance (Error/Correlation)	Limitations/Challenges	Validation Level
EKF (Extended Kalman Filter)	Estimation based on a linearized model of the vehicle	Mean error > 10%; correlation < 85%	Sensitivity to model errors and sudden load variations	Numerical simulation + vehicle test
RLS (Recursive Least Squares)	Iterative adjustment of parameters to reduce error	Modest accuracy	Instability in variable road conditions, noise sensitivity	
Artificial Neural Network (ANN) combined with fuzzy (static model)	Estimating mass and load distribution using AI and fuzzy logic	Error ~2.2 ÷ 3.9%; correlation > 91%	Requires large training datasets, complex calibration	
ANN combined with fuzzy and hardware-software 9-DOF model	Integration of static and dynamic tasks with real-time adaptation	Error < 8%, high stability in all tested scenarios	Computational complexity and industrial implementation still high	

**Table 7:** Synthesis of results regarding the identification of the transition between the adhesion and sliding regimes, obtained by mounting accelerometers in the tire structure, data from [95].

Investigated Parameter	Main Result	Impact on Vehicle Dynamics	Validation Level
Brake slip with vibrations indicating sliding	3.7% (full sliding at 13.7%)	Early detection of loss of traction, potential ABS input	Vehicle test
Toe angle ~1.5°	Measurable vibrations already at small slips	High sensitivity to geometric effects, relevance for ESC calibration	
Accelerometer signal stability	Correlation with steady-state force-slip characteristics	Supports identification of the grip–slip transition	

**Table 8:** Comparative overview of conditions for integrating deformation sensors into tire casing, data from [96].

Condition Investigated	Result	Interpretation	Impact on Vehicle Dynamics	Validation Level
Free rolling (FEM simulation)	Stable and periodic longitudinal deformation	Initial numerical validation for sensor	Allows calibration of longitudinal force generation models in free-running mode	Numerical simulation + vehicle test
Free rolling (physical test)	Similar experimental signal, lower amplitude (~1% peak strain, simulation values up to 5× higher)	Confirm the accuracy of the numerical prediction	Provides the actual database for wheel–road contact models in rolling-resistance estimation	
Braking (FEM simulation)	Deformation increases in the contact area	The model captures the transition to slipping	Direct relevance for ABS control	
Braking (physical test)	Signal with lower amplitude compared to simulation	Highlight the limitations of FEM prediction	Adjusting braking strategies for real road conditions	
Various vertical loads (FEM)	Increased deformation at high loads, tested at 3000 N, 3300 N, 3600 N	Confirm load sensitivity	Correlation between normal force and longitudinal-force generation	
Various vertical loads (physical test)	Trends similar to simulation, but with different amplitudes (10% load increase, 3% strain increase)	Experimental validation of sensor sensitivity	Allows control strategies to be adapted to variations in load	
Comparison between dry and snow-covered roads	Significantly increased amplitude on snow, loss of traction	The sensor clearly distinguishes road conditions	Important information for ESC systems and traction control algorithms in low-grip conditions	

**Table 9:** Factors influencing the amplitude of PVDF signals, data from [97].

Condition Investigated	Result	Impact on Vehicle Dynamics	Validation Level
Vertical load (1000 ÷ 5000 N)	PVDF signals increase proportionally with load	Direct correlation with normal force influencing lateral force generation	Numerical simulation + tire-rig experiment
Tire pressure (0.18 ÷ 0.26 MPa)	Modify the signal amplitude	Affects lateral stiffness and cornering stability	
Slip ratio (0 ÷ 16%)	Clear variations in signal	Relevance for ABS control and slip moment prediction	
Velocity (10 ÷ 50 km/h)	Sensitive response to speed increase	Scalability of the method at various dynamic regimes	
Tread wear (0 ÷ 4 mm)	Adjusts the sensitivity of the sensor	Requires adaptation of algorithms to maintain accuracy on worn tires	

**Table 10:** Summary of results regarding force measurement with a triaxial force sensor mounted on the tire belt beneath a tread block, data from [32].

Tested Condition	Longitudinal Load F_x_	Lateral Load F_y_	Vertical Load F_z_	Impact on Vehicle Dynamics	Validation Level
Vertical load (2000 ÷ 6000 N) at 0° slip angle, 10 km/h, 210 kPa	Variations proportional to slip	Moderate changes	Increases linearly with load	Accurate estimation of traction and stability	Tire-rig experiment
Slip angle (±1°) at 4000 N vertical load, 10 km/h, 210 kPa	Minor changes	High sensitivity	Almost constant	Critical relevance for lateral control and directional stability	

**Table 11:** Summary of results obtained using hybrid tire pressure monitoring systems, data from [59,100,101].

Research Conducted	Sensor Type Used	Tire-Wear Observations	Road-Surface Observations	Impact on Vehicle Dynamics	Validation Level
Velocity (20 ÷ 60 km/h)	Accelerometer	Greater amplitude, noise at >40 km/h	High-precision tri-axial acceleration signal	Robust surface and wear detection, ABS/ESC safety	Vehicle test + tire-rig experiment
Tire pressure (1.2 ÷ 2.7 bar)	PVDF and Accelerometer	High sensitivity to pressure variations	It changes the signal amplitude, but CNN maintains its position	Adaptive monitoring, grip optimization	
Vertical load (1 ÷ 6 kN)	Accelerometer and PVDF	Various distortions at high loads	Signals influenced, but CNN classification stable	Accurate estimation of normal forces, braking stability, and traction control	
Wear (level 1 ÷ 4/0 ÷ 100%)	PVDF	CNN classifies the degree of wear as robust	CNN maintains signal strength	Maintaining dynamic performance and long-term safety	
Track type	Accelerometer	-	CNN > LSTM, RF, SVM for real-time recognition	Precise classification of the road surface, ESP/ABS optimization	

**Table 12:** Summarized results for the friction-coefficient estimation method, data from [73].

Analyzed Parameter	Obtained Results	Impact on Vehicle Dynamics	Validation Level
Friction coefficient	RMSE 0.037 (proposed PF estimator) vs. 0.061 (EKF baseline)	Robust ABS/TCS control in mixed-grip conditions	Numerical simulation (real-time Hardware-in-the-Loop)
Longitudinal loads	RMSE 144.66 N (largest of the four wheels; wheel values 55.71 ÷ 144.66 N)	Optimization of brake and acceleration on each wheel	
Lateral load	RMSE 57.98 N (largest of the four wheels; wheel values 42.6 ÷ 57.98 N)	Improved lateral stability, increased maneuverability	
Slip angle	RMSE 0.077°	High predictability for ESC	
Algorithm (EKF)	Response time 535 ms (PF) vs. 567 ms (EKF)	Implementable in real time on automotive ECUs	

**Table 13:** Synthesis of the combined effects of camber angle, slip angle, and wheel load on the friction coefficient, data from [66].

Analyzed Parameter	Obtained Results	Impact on Vehicle Dynamics	Validation Level
Camber angle (0° ÷ 9°)	Increasing the camber angle leads to a decrease in contact-patch area, evaluated independently of friction effects (μ set to 0 in this analysis)	Progressive loss of lateral stability, risk of understeer when cornering	Numerical simulation + tire-rig experiment
Side slip angle (−10° ÷ +10°)	Asymmetric deformation of the contact patch and load redistribution	Changes directional stability	
Normal load (2000 ÷ 10,000 N)	The friction coefficient decreases as the normal load increases	Requires recalibration of ABS/ESC algorithms on loaded vehicles	
Contact-patch distribution	Becomes uneven at high values of the lateral slip angle and the angle of fall	Reduced comfort and accelerated tire wear	

**Table 14:** Cross-domain interdependency matrix linking structural parameters, sensor placement, estimation performance, and control boundaries for the three end-to-end cases discussed above (RFV, aquaplaning, low-adhesion TRFC), with implications for electric and autonomous vehicles.

Case	Structural Parameter	Sensor Placement	Estimation Performance	Control Boundary	EV/AV Impact
RFV	Radial stiffness non-uniformity [87]	Integrated deformation sensors [96]	Genetic algorithm/ANN-assisted structural optimization [88]	NVH/ride comfort threshold	Range, ride quality (EV)
Aquaplaning	Tread geometry, water film depth [29]	Real-time force monitoring [93]	Design-based delay on onset	ABS/ESC intervention timing	Trajectory safety (AV)
Low-adhesion TRFC	HT compound, lowest wet-grip indicator [13]	PVDF slip-angle sensing [97]	Estimation error 5.42% (dry), 8.89% (wet, μ = 0.4) [97]	ABS/ESC activation threshold	Perception redundancy (AV)

**Table 18:** Method-level traceability of dataset, scenario, metric, and limitation.

Method	Dataset	Scenario	Metric	Limitation	TRL
Particle filter (PF) [73]	Differential GPS (DGPS) + three low-cost inertial measurement units (IMUs); Hardware-in-the-Loop (HIL) on production-representative control hardware	Split-μ, sinusoidal steering	RMSE 0.037 (PF) vs. 0.061 (EKF); 535 ms vs. 567 ms	Robustness at constant speed/GPS-IMU loss	4 (HIL)
SVDKL [33]	Real electric-wheel vehicle, wet/dry asphalt	Constant acceleration/speed, small slip ratio	MAE 5.0%, RMSE 5.5%	Limited to wet/dry asphalt only	6
FEM + Flat-Trac/Calspan [66]	Flat-Trac (205/55R16, 10 km/h); Calspan Corporation (265/70R17, 60 km/h)	Slip up to ±24°, camber up to ±12° (values differ between the two tested tire sizes)	95 ÷ 97% accuracy	Two tire geometries only	5
PCKF-SOAFNN [62]	Real 4WID/4WIS EV test site	Lane-change + two novel 4WID/4WIS conditions	RMSE **↓** 21.80% (PCKF), **↓** 19.71% (SOAFNN)	Cannot handle complicated/bumpy roads	6
Transformer TNN [65]	IPG CarMaker (sim) + ZF WABCO, Dacia Duster SUV	Longitudinal/lateral maneuvers, multiple surfaces	MSE 1.5·10^−4^/1.7·10^−4^, RMSE ~0.012 ÷ 0.013	Black-box, no uncertainty; outliers at low excitation	6
Image + UKF fusion [103]	Withheld-from-training images, six real-world surfaces	Wet asphalt, waterlogged, muddy, post-snow, snow, rough/smooth ice	96.13% overall accuracy (all 6 conditions >95%)	Deliberately deflated tires used to test model mismatch	6
Camera + SVM fusion [104]	Real vehicle (SAIC Roewe), GPS base station	Wet tile road, 0 to 60 km/h	Fusion error ±0.05 vs. dynamics-only failure	Image processing dominates cost (0.1 ÷ 0.2 s/frame)	6
PVDF + GPR/MLP/RF [97]	FEM + physical test platform	Standard cross-validation; extreme maneuvers; tread wear 0 ÷ 4 mm	MAPE 5.47% to 7.16%; 8.31 ÷ 7.52% (wear)	Error > 20% at slip angles > 4° on wet	5
ST-SI-TC-IGA [90]	Near-actual geometry, CFD/structural sim	Rotating tire, road contact, deformation	No quantified accuracy metric reported	Purely computational, no physical validation	3
h-TPMS [100]	Real vehicle setup	32-min dry asphalt (baseline) + 32-min wet asphalt (robustness test)	Reliable 20% pressure-loss detection	Single sensor; index ≠1 at correct inflation	6
Intelligent tire prototype [96]	FEM (Abaqus) + Polaris GEM vehicle	Free-rolling, braking, acceleration; 3000 ÷ 3600 N loads	~1% peak strain; 10% load→3% strain	Simulated strain up to 5× higher than physical	6
Accelerometer braking [95]	Skid trailer + real vehicle, four tire sets	Full ABS braking maneuvers	Vibrations indicating sliding at 3.7% brake slip	Only four tire sets tested	6

## Data Availability

No new data were created or analyzed in this study. Data sharing is not applicable to this article.

## Abbreviations

The following abbreviations are used in this manuscript:

4WID	Four-Wheel Independent Driving
4WIS	Four-Wheel Independent Steering
ABS	Anti-Lock Braking System
AEB	Advanced Emergency Braking
ADAS	Advanced Driver Assistance Systems
AI	Artificial Intelligence
ANN	Artificial Neural Network
AV	Autonomous Vehicle
CAD	Computer-Aided Design
CAN	Controller Area Network
CFD	Computational Fluid Dynamics
CKF	Cubature Kalman Filter
CNN	Convolutional Neural Network
CWT	Continuous Wavelet Transform
DGPS	Differential Global Positioning System
DIC	Digital-Image Correlation
DLC	Dynamic-Load Coefficient
DOF	Degrees of Freedom
ECU	Electronic Control Unit
EKF	Extended Kalman Filter
EMC	Electromagnetic Compatibility
ESC	Electronic Stability Control
EV	Electric Vehicle
FBG	Fiber Bragg Grating
FEM	Finite-Element Method
GNSS	Global Navigation Satellite System
GPR	Gaussian Process Regression
GPS	Global Positioning System
GRU	Gated Recurrent Unit
HIL	Hardware-in-the-Loop
h-TPMS	Hybrid Tire-Pressure Monitoring System
HT	High-Temperature Processed Material
IGA	Isogeometric Analysis
IMM	Interacting Multiple Model
IMU	Inertial Measurement Unit
LCA	Life-Cycle Assessment
LiDAR	Light Detection and Ranging
LSTM	Long Short-Term Memory
MAE	Mean Absolute Error
MAPE	Mean Absolute Percentage Error
MEMS	Micro-Electro-Mechanical Systems
MLP	Multi-Layer Perceptron
MPCA	Model Predictive Control Allocation
NHTSA	National Highway Traffic Safety Administration
NVH	Noise, Vibration, Harshness
OEM	Original Equipment Manufacturer
PF	Particle Filter
PCKF	Prior Knowledge-Based Cubature Kalman Filter
PMI	Proportional-Multiple-Integral
PRISMA	Preferred Reporting Items for Systematic Reviews and Meta-Analyses
PSTCNN	Parallel Spatio-Temporal Convolutional Neural Network
PVDF	Polyvinylidene Fluoride
RF	Random Forest
RFV	Radial-Force Variation
RLS	Recursive Least Squares
RMSE	Root Mean Squared Error
SI	Slip Interface
SVDKL	Stochastic Variational Deep Kernel Learning
SVGP	Sparse Variational Gaussian Process
SOAFNN	Self-Organizing Asymmetric Fuzzy Neural Network
STPB	Silane-Terminated Telechelic Polybutadiene
ST	Space-Time
SVM	Support Vector Machines
TC	Topology Change
TDAE	Treated Distillate Aromatic Extract
TNN	Transformer Neural Network
TPMS	Tire Pressure Monitoring System
TRL	Technology Readiness Level
TRFC	Tire–Road Friction Coefficient
TWUO	Tire Workload Usage Optimization
UKF	Unscented Kalman Filter
VDA	Verband der Automobilindustrie

## Appendix A. Database-Specific Search String, Filters, and Retrieved Records

**Table A1:** Database-specific search strings, filters, and retrieved records.

Database	Search String	Filter Applied	Records
ScienceDirect	(“smart tire” OR “intelligent tire” OR “predictive tire” OR “tire–road friction” OR “TRFC estimation” OR “tire sensor” OR “PVDF tire” OR “FBG tire”) AND (“electric vehicle” OR “autonomous vehicle”)	2014–2026; research articles	1065
Web of Science (Core Collection)	(“smart tire” OR “intelligent tire” OR “predictive tire” OR “tire–road friction” OR “TRFC estimation” OR “tire sensor” OR “PVDF tire” OR “FBG tire”)	All Fields; Editions: All; 2014–2026	80
IEEE Xplore	(“All Metadata”: smart tire) OR (“All Metadata”: intelligent tire) OR (“All Metadata”: predictive tire) OR (“All Metadata”: tire–road friction) OR (“All Metadata”: TRFC estimation) OR (“All Metadata”: tire sensor) OR (“All Metadata”: FBG tire)	2014–2026; journal articles and early-access articles only (conference proceedings excluded)	1185
		Total records identified	2330

## References

[1] Deng Y., Wang Z., Liu T., Liang W., Shen H., Xiao Z. (2023). Static and Dynamic Mechanical Characteristics of Honeycomb Non-Pneumatic Tire under Structural Damage Condition. Eur. J. Mech.-A/Solids.

[2] Fu H., Liang X., Chen K., Wang Y., Xiao Z. (2022). Study on Key Mechanical Properties of the Flexible Spoke Non-Pneumatic Tire Considering Thermo-Mechanical Coupling. Adv. Eng. Softw..

[3] Shuai Z., Gao S., Yu Y., Zhao X., Yang W., Song Z., Zheng Y., Yuan J., Jiao Z. (2024). Characteristic Study and Design Factor Analysis of a Novel Non-Pneumatic Tyre with V-Shaped Spokes. Mater. Des..

[4] Yang L., Yue M., Liu Y., Guo L. (2020). RBFNN Based Terminal Sliding Mode Adaptive Control for Electric Ground Vehicles after Tire Blowout on Expressway. Appl. Soft Comput..

[5] Zang L., Xue C., Peng X., Jiao J., Feng Y., Mao Y. (2024). Investigation on Dynamic Characteristics of Inserts Supporting Run-Flat Tyre Based on the Modified Non-Linear Ground Pressure Distribution. Int. J. Non-Linear Mech..

[6] Deng Y., Wang Z., Shen H., Gong J., Xiao Z. (2023). A Comprehensive Review on Non-Pneumatic Tyre Research. Mater. Des..

[7] Do J., Hyun D., Han K., Choi S.B. (2024). Real-Time Estimation of Longitudinal Tire Stiffness Considering Dynamic Characteristics of Tire. Mechatronics.

[8] Salari A.H., Mirzaeinejad H., Mahani M.F. (2023). Tire Normal Force Estimation Using Artificial Neural Networks and Fuzzy Classifiers: Experimental Validation. Appl. Soft Comput..

[9] Wang Y., Liu M., Chen Y. (2022). Safety-Guaranteed Control of Coupled Longitudinal and Lateral Vehicle Dynamics by Considering Tire Stability via CDBFs. IFAC-PapersOnLine.

[10] Sangiuliano L., Reff B., Palandri J., Wolf-Monheim F., Pluymers B., Deckers E., Desmet W., Claeys C. (2022). Low Frequency Tyre Noise Mitigation in a Vehicle Using Metal 3D Printed Resonant Metamaterials. Mech. Syst. Signal Process..

[11] Zhang M., Unrau H.-J., Gießler M., Gauterin F. (2024). A Detailed Tire Tread Friction Model Considering Dynamic Friction States. Tribol. Int..

[12] Ammari O., El Majdoub K., Giri F., Baz R. (2024). Modeling and Control Design for Half Electric Vehicle with Wheel BLDC Actuator and Pacejka’s Tire. Comput. Electr. Eng..

[13] Lim K., Hong S.J., Lee H., Jung J., Kim K., Park J.H., Bang J. (2024). Open-Structured Silica Network Based on Silane-Terminated Telechelic Polybutadiene for Electric Vehicle Tires. Sustain. Mater. Technol..

[14] Lin F., Qian C., Cai Y., Zhao Y., Wang S., Zang L. (2022). Integrated Tire Slip Energy Dissipation and Lateral Stability Control of Distributed Drive Electric Vehicle with Mechanical Elastic Wheel. J. Frankl. Inst..

[15] Sakhnevych A., Genovese A. (2024). Tyre Wear Model: A Fusion of Rubber Viscoelasticity, Road Roughness, and Thermodynamic State. Wear.

[16] Wang Q., Li A., Yan J. (2025). Study on the Relationship between the Steady-State Rolling Resistance of Radial Tires and Energy Consumption of Electric Vehicles. Sci. Rep..

[17] Lolage M., Parida P., Gupta A., Rautaray D. (2022). Synergistic Effects of Silica and Nanoclay on Curing Characteristics, Processing Behaviour and Mechanical Properties of Solution Styrene Butadiene Rubber (SBR)–Based Tire Tread Compounds. Emergent Mater..

[18] Yang S., Chen Y., Shi R., Wang R., Cao Y., Lu J. (2022). A Survey of Intelligent Tires for Tire-Road Interaction Recognition Toward Autonomous Vehicles. IEEE Trans. Intell. Veh..

[19] Wang Y., Hu J., Wang F., Dong H., Yan Y., Ren Y., Zhou C., Yin G. (2022). Tire Road Friction Coefficient Estimation: Review and Research Perspectives. Chin. J. Mech. Eng..

[20] Pomoni M. (2022). Exploring Smart Tires as a Tool to Assist Safe Driving and Monitor Tire–Road Friction. Vehicles.

[21] Zhang Q., Wang J., Sun N., Peng X., Pi D., Yin G. (2025). Tire Pressure Monitoring Methods for Vehicles: Review and Research Perspectives. Measurement.

[22] Wu T., Zhang X., Wang D., Zhang W., Pan D., Tao L. (2025). Design and Implementation of Novel Testing System for Intelligent Tire Development: From Bench to Road. Sensors.

[23] Zhang X., Göhlich D. (2017). A Hierarchical Estimator Development for Estimation of Tire-Road Friction Coefficient. PLoS ONE.

[24] Berge T., Mioduszewski P., Hałucha M., Bohatkiewicz J. (2025). ISO Test Track Influence on the EU Tyre Label Noise Value. Appl. Acoust..

[25] Park H., Seo J., Kim K., Kim T. (2024). Predicting the Wear Amount of Tire Tread Using 1D−CNN. Sensors.

[26] Prasshanth C.V., Sugumaran V. (2024). Tire Wear Monitoring Using Feature Fusion and CatBoost Classifier. Artif. Intell. Rev..

[27] National Highway Traffic Safety Administration (2012). Tire-Related Factors in the Pre-Crash Phase.

[28] D’Alessandro V., Melzi S., Sbrosi M., Brusarosco M. (2012). Phenomenological Analysis of Hydroplaning through Intelligent Tyres. Veh. Syst. Dyn..

[29] Maleska M., Petry F., Fehr D., Schuhmann W., Böhle M. (2021). Longitudinal Hydroplaning Performance of Passenger Car Tires. Veh. Syst. Dyn..

[30] Zhu X., Pang Y., Yang J., Zhao H. (2022). Numerical Analysis of Hydroplaning Behaviour by Using a Tire–Water-Film–Runway Model. Int. J. Pavement Eng..

[31] Shin W.S., Kwon Y.R., Kim J.S., Hong S.J., Kim Y.J., Lim S.H., Chang Y.W., Kim D.H. (2021). Improved Silica Dispersibility in Silica-Rubber Compounds for a Tire Tread by Using an Itaconic Acid-Based Polymeric Dispersant. Fibers Polym..

[32] Matsubara M., Toyoshima T., Shishido H., Tajiri D., Kawamura S. (2024). Measurement of the Three-Directional Contact Force Using a Smart Tire with a Force Sensor. Veh. Syst. Dyn..

[33] Chen L., Qin Z., Bian Y., Hu M., Peng X. (2025). Data-Driven Tire-Road Friction Estimation for Electric-Wheel Vehicle with Data Category Selection and Uncertainty Evaluation. IEEE Trans. Ind. Electron..

[34] Matilainen M., Tuononen A. (2015). Tyre Contact Length on Dry and Wet Road Surfaces Measured by Three-Axial Accelerometer. Mech. Syst. Signal Process..

[35] Ejsmont J., Ronowski G., Ydrefors L., Owczarzak W., Sommer S., Świeczko-Żurek B. (2024). Comparison of Tire Rolling Resistance Measuring Methods for Different Surfaces. Int. J. Automot. Technol..

[36] Erdogan G., Hong S., Borrelli F., Hedrick K. (2011). Tire Sensors for the Measurement of Slip Angle and Friction Coefficient and Their Use in Stability Control Systems. SAE Int. J. Passeng. Cars–Mech. Syst..

[37] Quan Z., Li B., Bei S., Sun X., Xu N., Gu T. (2023). Tire-Road Friction Coefficient Estimation Method Design for Intelligent Tires Equipped with PVDF Piezoelectric Film Sensors. Sens. Actuators A Phys..

[38] Baffet G., Charara A., Lechner D. (2009). Estimation of Vehicle Sideslip, Tire Force and Wheel Cornering Stiffness. Control Eng. Pract..

[39] Carcaterra A., Roveri N. (2013). Tire Grip Identification Based on Strain Information: Theory and Simulations. Mech. Syst. Signal Process..

[40] Chen X., Li S., Li L., Zhao W., Cheng S. (2022). Longitudinal-Lateral-Cooperative Estimation Algorithm for Vehicle Dynamics States Based on Adaptive-Square-Root-Cubature-Kalman-Filter and Similarity-Principle. Mech. Syst. Signal Process..

[41] Cordeiro R.A., Victorino A.C., Ferreira P.A.V., De Paiva E.C., Bueno S.S. (2016). Tire-Ground Forces Estimation in a 4-Wheel Vehicle Using a Delayed Interconnected Cascade-Observer Structure**The Authors Acknowledge the Grants: Ph.D. FAPESP (2014/06610-8), BEPE FAPESP (2014/27240-4) and Regular FAPESP Project VERDE (2014/02672-9). This Work Was Carried out within the Framework of the Equipex ROBOTEX (Reference ANR-10-EQPX-44-01) Heudiasyc Laboratory UMR CNRS UTC 7253. IFAC-PapersOnLine.

[42] Mendoza-Petit M.F., Garcia-Pozuelo D., Diaz V., Garrosa M. (2022). Characterization of the Loss of Grip Condition in the Strain-Based Intelligent Tire at Severe Maneuvers. Mech. Syst. Signal Process..

[43] Wan W., Sheng K., Cai Q., Ao L., Ouyang N., Feng H., Sime D.M. (2025). Correction to: Enhanced Tire Road Friction Coefficient Estimation through Interacting Multiple Model Design Based on Tire Force Observation. Nonlinear Dyn..

[44] Yang Z., Linhui Z. (2025). Distributed Nonlinear Estimation of Vehicle States and Tire Forces with Stability Guarantees. Control Eng. Pract..

[45] Yang H., Cheng H., Zuo Z., Li H. (2022). ESO-Based Lateral Control for Electrical Vehicles with Unmodeled Tire Dynamics on Uneven Road. Mech. Syst. Signal Process..

[46] Dwivedi C., Mohanty T.R., Manjare S., Ramakrishnan S., Amarnath S.K.P., Lorenzetti D. (2025). Advanced Carbon-Silica Hybrid Fillers for Enhanced Rubber Compounds in Tires. Emergent Mater..

[47] Neethirajan J., Parathodika A.R., Hu G.-H., Naskar K. (2022). Functional Rubber Composites Based on Silica-Silane Reinforcement for Green Tire Application: The State of the Art. Funct. Compos. Mater..

[48] Danci C.C., Duma I., Buidin T.I.C., Cordoș N., Todoruț A. (2024). Vertical Vibration Analysis of a Formula Student Vehicle Using Different Suspension System Configurations. Acta Tech. Napoc..

[49] (2018). Passenger Car, Truck and Bus Tyre Rolling Resistance Measurement Method—Single Point Test and Correlation of Measurement Results.

[50] Zhao B., Xu N., Chen H., Guo K., Huang Y. (2019). Stability Control of Electric Vehicles with In-Wheel Motors by Considering Tire Slip Energy. Mech. Syst. Signal Process..

[51] Lian Y.F., Wang X.Y., Zhao Y., Tian Y.T. (2015). Direct Yaw-Moment Robust Control for Electric Vehicles Based on Simplified Lateral Tire Dynamic Models and Vehicle Model. IFAC-PapersOnLine.

[52] Ni J., Hu J., Xiang C. (2017). Relaxed Static Stability Based on Tyre Cornering Stiffness Estimation for All-Wheel-Drive Electric Vehicle. Control Eng. Pract..

[53] Siramdasu Y., Taheri S. (2016). Discrete Tyre Model Application for Evaluation of Vehicle Limit Handling Performance. Veh. Syst. Dyn..

[54] Wang Q., Zhao Y., Xie W., Zhao Q., Lin F. (2022). Hierarchical Estimation of Vehicle State and Tire Forces for Distributed In-Wheel Motor Drive Electric Vehicle without Previously Established Tire Model. J. Frankl. Inst..

[55] Wang R., Hu C., Wang Z., Yan F., Chen N. (2015). Integrated Optimal Dynamics Control of 4WD4WS Electric Ground Vehicle with Tire-Road Frictional Coefficient Estimation. Mech. Syst. Signal Process..

[56] Wang R., Zhang H., Wang J., Yan F., Chen N. (2015). Robust Lateral Motion Control of Four-Wheel Independently Actuated Electric Vehicles with Tire Force Saturation Consideration. J. Frankl. Inst..

[57] Zhang H., Xv K., Li Y., Zhao W., Wang C. (2024). Coordination Control Strategy of Motion Stability and Tire Slip for Electric Vehicle Driven by In-Wheel-Motors. J. Frankl. Inst..

[58] Feng Y., Chen H., Zhao H., Zhou H. (2020). Road Tire Friction Coefficient Estimation for Four Wheel Drive Electric Vehicle Based on Moving Optimal Estimation Strategy. Mech. Syst. Signal Process..

[59] Lee D., Kim J.-C., Kim M., Lee H. (2021). Intelligent Tire Sensor-Based Real-Time Road Surface Classification Using an Artificial Neural Network. Sensors.

[60] Li M., Jia Y., Lei T. (2024). Path Tracking of Varying-Velocity 4WS Autonomous Vehicles under Tire Force Friction Ellipse Constraints. Robot. Auton. Syst..

[61] Maurya D., Kumar P., Khaleghian S., Sriramdas R., Kang M.G., Kishore R.A., Kumar V., Song H.-C., Park J.-M. (Jerry), Taheri S. (2018). Energy Harvesting and Strain Sensing in Smart Tire for next Generation Autonomous Vehicles. Appl. Energy.

[62] Zhang G., Wang X., Li L., Zhao X. (2024). Tire–Road Friction Estimation for Four-Wheel Independent Steering and Driving EVs Using Improved CKF and FNN. IEEE Trans. Transp. Electrif..

[63] He Y., Yang X., Xiao S., Liu P., Lu G., Xing C., Fan Z., Sheng W., Wang D. (2023). Experimental Study on the High-Speed Frictional Behavior between the Tire and Asphalt Pavement. Constr. Build. Mater..

[64] Hashemi E., Pirani M., Khajepour A., Kasaiezadeh A., Chen S.-K., Litkouhi B. (2017). Corner-Based Estimation of Tire Forces and Vehicle Velocities Robust to Road Conditions. Control Eng. Pract..

[65] Schäfke H., Lampe N., Kortmann K.-P. (2023). Transformer Neural Networks for Maximum Friction Coefficient Estimation of Tire-Road Contact Using Onboard Vehicle Sensors. Proceedings of the 2023 62nd IEEE Conference on Decision and Control (CDC).

[66] Suo Y., Lu D., Wu H., Zhang Y., Zhou T. (2024). Tire-Road Friction Coefficient Estimation of Sideslip-Camber Combined Situation. Proceedings of the 2024 IEEE 99th Vehicular Technology Conference (VTC2024-Spring).

[67] Tao S., Ju Z., Li L., Zhang H., Pedrycz W. (2024). Tire Road Friction Coefficient Estimation for Individual Wheel Based on Two Robust PMI Observers and a Multilayer Perceptron. IEEE Trans. Veh. Technol..

[68] Ge H., Quezada J.C., Houerou V.L., Chazallon C. (2022). Multiscale Analysis of Tire and Asphalt Pavement Interaction via Coupling FEM–DEM Simulation. Eng. Struct..

[69] Page M.J., McKenzie J.E., Bossuyt P.M., Boutron I., Hoffmann T.C., Mulrow C.D., Shamseer L., Tetzlaff J.M., Akl E.A., Brennan S.E. (2021). The PRISMA 2020 Statement: An Updated Guideline for Reporting Systematic Reviews. BMJ.

[70] (2018). Road Vehicles—Functional Safety.

[71] Tan R., Ye S., Peng Q., Du C., Zhou Z. (2025). Research on Efficient Prediction and Suppression of Electromagnetic Interference in Electric Drive Systems. World Electr. Veh. J..

[72] European Commission (2014). Horizon 2020 Work Programme 2014–2015, General Annexes, Annex G: Technology Readiness Levels (TRL).

[73] Anupam K., Srirangam S.K., Scarpas A., Kasbergen C., Kane M. (2014). Study of Cornering Maneuvers of a Pneumatic Tire on Asphalt Pavement Surfaces Using the Finite Element Method. Transp. Res. Rec..

[74] Lee S.-K., Yoo J., Lee C.-H., An K., Yoon Y.-S., Lee J., Yeom G.-H., Hwang S.-U. (2023). Road Type Classification Using Deep Learning for Tire-Pavement Interaction Noise Data in Autonomous Driving Vehicle. Appl. Acoust..

[75] Venturini S., Bonisoli E., Rosso C., Velardocchia M. (2024). Corrigendum to “A Tyre-Rim Interaction Digital Twin for Biaxial Loading Conditions” [Mech. Mach. Theory 2024, 191, 105491]. Mech. Mach. Theory.

[76] Cha H., Kim J., Yi K., Park J. (2024). Investigation into High-Performance Tire Characteristics at the Limits of Handling. Veh. Syst. Dyn..

[77] Guo N., Zhang X., Zou Y., Lenzo B., Zhang T., Göhlich D. (2020). A Fast Model Predictive Control Allocation of Distributed Drive Electric Vehicles for Tire Slip Energy Saving with Stability Constraints. Control Eng. Pract..

[78] Suzuki Y., Kano Y., Abe M. (2014). A Study on Tyre Force Distribution Controls for Full Drive-by-Wire Electric Vehicle. Veh. Syst. Dyn..

[79] Kobayashi T., Katsuyama E., Sugiura H., Ono E., Yamamoto M. (2017). Direct Yaw Moment Control and Power Consumption of In-Wheel Motor Vehicle in Steady-State Turning. Veh. Syst. Dyn..

[80] Zhao H., Ren B., Chen H., Deng W. (2015). Model Predictive Control Allocation for Stability Improvement of Four-wheel Drive Electric Vehicles in Critical Driving Condition. IET Control Theory Appl..

[81] Chen X., Sathyanarayan H., Gong Y., Yi J., Wang H. (2023). Dynamic Tire/Road Friction Estimation with Embedded Flexible Force Sensors. IEEE Sens. J..

[82] Liu W., Wang R., Ding R., Meng X., Yang L. (2020). On-Line Estimation of Road Profile in Semi-Active Suspension Based on Unsprung Mass Acceleration. Mech. Syst. Signal Process..

[83] Towards the Prediction of Hydroplaning: Numerical Simulation and Experimental Validation (TROPHY). https://cordis.europa.eu/project/id/G3RD-CT-2001-00510/factsheet.

[84] Heißing B., Ersoy M., Gies S. (2013). Fahrwerkhandbuch: Grundlagen, Fahrdynamik, Komponenten, Systeme, Mechatronik, Perspektiven.

[85] Gunreddy P.R., Leamy M. Split Ring Resonator-Based Metamaterial with Total Bandgap for Reducing NVH in Electric Vehicles. Proceedings of the WCX SAE World Congress Experience 2024.

[86] Opala M. (2025). Evaluation of Ride Quality in a Passenger Car with Non-Uniform Tyre Radial Stiffness. Veh. Syst. Dyn..

[87] Cho J.R., Lee J.H. (2017). Multi-Objective Optimum Design of TBR Tire Structure for Enhancing the Durability Using Genetic Algorithm. J. Mech. Sci. Technol..

[88] Liu M., Li Z., Wang Y. (2024). Experimental and Numerical Investigation on Radial Impact of Pneumatic Tyre. Int. J. Impact Eng..

[89] Kuraishi T., Xu Z., Takizawa K., Tezduyar T.E., Kakegami T. (2025). Correction to: Space–Time Isogeometric Analysis of Tire Aerodynamics with Complex Tread Pattern, Road Contact, and Tire Deformation. Comput. Mech..

[90] Bernal-Ortega P., Van Elburg F., Araujo-Morera J., Gojzewski H., Blume A. (2024). Heading towards a Fully Sustainable Tire Tread Compound: Use of Bio-Based Resins. Polym. Test..

[91] Jung J., Sodano H.A. (2022). Cellulose Nanocrystal Functionalized Aramid Nanofiber Reinforced Rubber Compounds for Tire Tread Application. Cellulose.

[92] Zhao W., Zhang C., Zhang J. (2020). Continuous Measurement of Tire Deformation Using Long-Gauge Strain Sensors. Mech. Syst. Signal Process..

[93] Gu T., Li B., Quan Z., Bei S., Yin G., Guo J., Zhou X., Han X. (2022). The Vertical Force Estimation Algorithm Based on Smart Tire Technology. WEVJ.

[94] Degenhart F., Edelmann J., Plöchl M. (2021). Application of Tire Sensors for an Integrated Approach to Evaluate Longitudinal Tire Characteristics for Braking Performance. Proc. Inst. Mech. Eng. Part D J. Automob. Eng..

[95] Bastiaan J., Chawan A., Eum W., Alipour K., Rouhollahi F., Behroozi M., Baqersad J. (2024). Intelligent Tire Prototype in Longitudinal Slip Operating Conditions. Sensors.

[96] Sun X., Quan Z., Cai Y., Chen L., Li B. (2025). Direct Tire Slip Angle Estimation Using Intelligent Tire Equipped with PVDF Sensors. IEEE/ASME Trans. Mechatron..

[97] United Nations Economic Commission for Europe (2010). Uniform Provisions Concerning the Approval of Vehicles with Regard to Their Equipment Which May Include a Tyre Pressure Monitoring System.

[98] United Nations Economic Commission for Europe (2022). Uniform Provisions Concerning the Approval of Vehicles of Categories M2, M3, N and O with Regard to Tyre Pressure Monitoring Systems.

[99] Formentin S., Onesto L., Colombo T., Pozzato A., Savaresi S.M. (2021). H-TPMS: A Hybrid Tire Pressure Monitoring System for Road Vehicles. Mechatronics.

[100] Tong Z., Cao Y., Wang R., Chen Y., Li Z., Lu J., Yang S. (2025). Machine Learning-Driven Intelligent Tire Wear Detection System. Measurement.

[101] Zhao J., Du J., Zhu B., Luo X., Tao X. (2023). Indirect Tire Pressure Monitoring Method Based on the Fusion of Time and Frequency Domain Analysis. Measurement.

[102] Zhang L., Guo P., Wang Z., Ding X. (2023). An Enabling Tire-Road Friction Estimation Method for Four-in-Wheel-Motor-Drive Electric Vehicles. IEEE Trans. Transp. Electrif..

[103] Zhao S., Zhang J., Jiang Y., He C., Han J. (2025). Tire-Road Friction Coefficients Adaptive Estimation through Image and Vehicle Dynamics Integration. Mech. Syst. Signal Process..

[104] Leng B., Jin D., Xiong L., Yang X., Yu Z. (2021). Estimation of Tire-Road Peak Adhesion Coefficient for Intelligent Electric Vehicles Based on Camera and Tire Dynamics Information Fusion. Mech. Syst. Signal Process..

[105] Huang J., Labi S., Kulcsár B.A., Gao Y., Monreal C.O., Wu F., He Z., Qu X. (2024). Intelligent Vehicles and Smart Transportation. Fundam. Res..

[106] Yue Y., Li X., Zhao Z., Wang H., Guo X. (2023). Stretchable Flexible Sensors for Smart Tires Based on Laser-Induced Graphene Technology.

[107] Karkaria V., Chen J., Luey C., Siuta C., Lim D., Radulescu R. (2025). A Digital Twin Framework Utilizing Machine Learning for Robust Predictive Maintenance: Enhancing Tire Health Monitoring. J. Comput. Inf. Sci. Eng..

